# Egg consumption and risk of cardiovascular disease: three large prospective US cohort studies, systematic review, and updated meta-analysis

**DOI:** 10.1136/bmj.m513

**Published:** 2020-03-04

**Authors:** Jean-Philippe Drouin-Chartier, Siyu Chen, Yanping Li, Amanda L Schwab, Meir J Stampfer, Frank M Sacks, Bernard Rosner, Walter C Willett, Frank B Hu, Shilpa N Bhupathiraju

**Affiliations:** 1 Department of Nutrition, Harvard T H Chan School of Public Health, 677 Huntington Avenue, Boston, MA 02115, USA; 2 Department of Epidemiology, Harvard T H Chan School of Public Health, Boston, MA, USA; 3 Channing Division of Network Medicine, Department of Medicine, Brigham and Women’s Hospital, Harvard Medical School, Boston, MA, USA; 4 Department of Biostatistics, Harvard T H Chan School of Public Health, Boston, MA, USA

## Abstract

**Objective:**

To evaluate the association between egg intake and cardiovascular disease risk among women and men in the United States, and to conduct a meta-analysis of prospective cohort studies.

**Design:**

Prospective cohort study, and a systematic review and meta-analysis of prospective cohort studies.

**Setting:**

Nurses’ Health Study (NHS, 1980-2012), NHS II (1991-2013), Health Professionals’ Follow-Up Study (HPFS, 1986-2012).

**Participants:**

Cohort analyses included 83 349 women from NHS, 90 214 women from NHS II, and 42 055 men from HPFS who were free of cardiovascular disease, type 2 diabetes, and cancer at baseline.

**Main outcome measures:**

Incident cardiovascular disease, which included non-fatal myocardial infarction, fatal coronary heart disease, and stroke.

**Results:**

Over up to 32 years of follow-up (>5.54 million person years), 14 806 participants with incident cardiovascular disease were identified in the three cohorts. Participants with a higher egg intake had a higher body mass index, were less likely to be treated with statins, and consumed more red meats. Most people consumed between one and less than five eggs per week. In the pooled multivariable analysis, consumption of at least one egg per day was not associated with incident cardiovascular disease risk after adjustment for updated lifestyle and dietary factors associated with egg intake (hazard ratio for at least one egg per day *v* less than one egg per month 0.93, 95% confidence interval 0.82 to 1.05). In the updated meta-analysis of prospective cohort studies (33 risk estimates, 1 720 108 participants, 139 195 cardiovascular disease events), an increase of one egg per day was not associated with cardiovascular disease risk (pooled relative risk 0.98, 95% confidence interval 0.93 to 1.03, I^2^=62.3%). Results were similar for coronary heart disease (21 risk estimates, 1 411 261 participants, 59 713 coronary heart disease events; 0.96, 0.91 to 1.03, I^2^=38.2%), and stroke (22 risk estimates, 1 059 315 participants, 53 617 stroke events; 0.99, 0.91 to 1.07, I^2^=71.5%). In analyses stratified by geographical location (P for interaction=0.07), no association was found between egg consumption and cardiovascular disease risk among US cohorts (1.01, 0.96 to 1.06, I^2^=30.8%) or European cohorts (1.05, 0.92 to 1.19, I^2^=64.7%), but an inverse association was seen in Asian cohorts (0.92, 0.85 to 0.99, I^2^=44.8%).

**Conclusions:**

Results from the three cohorts and from the updated meta-analysis show that moderate egg consumption (up to one egg per day) is not associated with cardiovascular disease risk overall, and is associated with potentially lower cardiovascular disease risk in Asian populations.

**Systematic review registration:**

PROSPERO CRD42019129650.

## Introduction

In the United States, cardiovascular disease is the leading cause of death in men and women.^1^ Diet and lifestyle undisputedly play a major part in the development of cardiovascular disease.^2^ In the past, limiting dietary cholesterol intake to 300 mg per day was widely recommended to prevent cardiovascular disease.^2^ However, because of the weak association between dietary cholesterol and blood cholesterol, and considering that dietary cholesterol is no longer a nutrient of concern for overconsumption, the most recent 2015 dietary guidelines for Americans did not carry forward this recommendation.^3^

Eggs are a major source of dietary cholesterol, but they are also an affordable source of high quality protein, iron, unsaturated fatty acids, phospholipids, and carotenoids.^4^ However, because of the cholesterol content in eggs, the association between egg intake and cardiovascular disease risk has been a topic of intense debate in the past decade. Many prospective studies on the association between egg intake and cardiovascular disease risk have provided conflicting findings. Some studies have reported no association between egg intake and risk of cardiovascular disease,^5^ ^6^ ^7^ some have reported a higher risk,^8^ ^9^ and others have reported an inverse association with cardiovascular disease events^10^ ^11^ or subclinical measures.^12^ Even meta-analyses of prospective studies on egg consumption and cardiovascular disease risk did not provide consistent results and created further confusion.^13^ ^14^ ^15^ ^16^ ^17^

To evaluate the association between egg intake and cardiovascular disease risk, it is desirable to have repeated measures of diet and lifestyle. Such measures account for random variation in intake, provide a measure of long term or usual diet, and sufficiently account for confounding owing to lifestyle factors because atherosclerosis develops over many decades. Additionally, to inform dietary guidelines while also considering sustainability challenges associated with food production, it is critical to estimate the effects on cardiovascular disease risk of eggs compared with other animal and plant based foods.^18^ In our study, we examined the association between egg intake and incident cardiovascular disease by using repeated measures of diet over up to 32 years of follow-up with detailed control of dietary and other potential confounders. Our analyses included men and women from three large US cohorts: the Nurses’ Health Study (NHS), NHS II, and the Health Professionals’ Follow-Up Study (HPFS). Our study is an updated analysis of the study published in 1999 by Hu and colleagues^7^ and includes up to 24 additional years of follow-up, more than 10 times the number of events, and extends the analysis to the younger cohort of NHS II. We also used these data to statistically estimate how replacing eggs with other foods is associated with cardiovascular disease risk. Finally, we conducted an updated systematic review of the literature and meta-analysis of egg consumption and cardiovascular disease risk that include these new results.

## Methods

### Cohort analyses

#### Study population

NHS, NHS II, and HPFS are prospective cohort studies of US health professionals. In 1976, NHS enrolled 121 701 female registered nurses between the ages of 30 and 55 years to investigate the long term health effects of various contraceptive methods in women.^19^ ^20^ NHS II includes 116 430 female registered nurses between the ages of 25 and 44 years and began in 1989 with the goal of investigating oral contraceptives, diet, and lifestyle factors in a population younger than NHS.^20^ HPFS is the male counterpart to NHS and NHS II, and included 51 529 male health professionals between the ages of 40 and 75 years at study inception in 1986. In all three cohorts, participants completed validated questionnaires every two years that captured information on disease diagnosis, disease risk factors, drug use, and lifestyle characteristics, with follow-up rates exceeding 90%. For the current analysis, baseline was defined as the year when diet was first assessed in the cohorts: 1980 in NHS, 1986 in HPFS, and 1991 in NHS II. Of the participants who completed a baseline food frequency questionnaire (NHS, n=98 047; NHS II, n=97 813; HPFS, n=51 529), we excluded those who reported a diagnosis of cardiovascular disease, cancer, or type 2 diabetes, or had coronary artery surgery before completion of the baseline; these diagnoses could result in changes in diet.^21^ We also excluded participants with missing age at baseline and those who reported implausible energy intake on the food frequency questionnaire (<500 or >3500 kcal/day for women and <800 or >4200 kcal/day for men). Additionally, we excluded participants who left more than 70 items blank on the food frequency questionnaire, and those who had missing information on baseline egg intake. The final sample included 83 349 participants in NHS, 90 214 participants in NHS II, and 42 055 participants in HPFS. Supplemental figure 1 shows the flow chart of participants.

The institutional review board at the Brigham and Women’s Hospital and Harvard T.H. Chan School of Public Health approved the study protocol, with informed consent indicated by the return of the baseline questionnaire.

#### Assessment of egg intake

Whole egg intake was reported every two to four years, beginning in 1980, 1991, and 1986 for NHS, NHS II, and HPFS, respectively, by using a validated semiquantitative food frequency questionnaire. The questionnaire had 61 items in 1980 and 126-131 items in subsequent versions.^22^ ^23^ ^24^ Participants were asked how often on average they consumed whole eggs with yolk in the past year. Reported intake excluded eggs in baked goods (eg, cake), liquid eggs, and egg whites. Among 173 women in NHS, food frequency questionnaires were validated against four seven day weighed diet records. The deattenuated correlation coefficient between the food frequency questionnaire and the weighed food record for whole egg intake was 0.77.^25^ A similar validation study conducted among 127 men in HPFS showed a high correlation between whole egg intake reported from food frequency questionnaires and weighed food records (deattenuated correlation coefficient=0.80).^26^ Consumption of liquid eggs and egg whites was not assessed in the questionnaire. However, we computed intake of eggs included in baked goods such as cakes, cookies, pancakes, muffins, sweet rolls, and donuts.

In the main analyses, we used whole egg intake as the exposure. In a sensitivity analysis, we used total egg consumption (consumption of whole eggs plus eggs in baked goods) as the exposure.

#### Assessment of incident cardiovascular disease

The primary endpoint for this study was incident cardiovascular disease, which we defined as non-fatal myocardial infarction, fatal coronary heart disease, and fatal and non-fatal stroke that occurred after baseline. Participants (or next of kin for deceased) who reported a primary endpoint were asked for permission to have their medical records reviewed by study physicians who were blinded to the participants’ exposure and risk factor status. We used World Health Organization criteria of typical symptoms plus either elevated enzymes (including troponin) or diagnostic electrocardiographic findings to diagnose myocardial infarction.^27^ Stroke was confirmed using criteria from the National Survey of Stroke, which required evidence of a neurological deficit with sudden or rapid onset that persisted for more than 24 hours or until death.^28^ A physician reviewed stroke events and classified them by stroke subtype or cause using the Perth Community Stroke Study criteria (subarachnoid hemorrhage, intraparenchymal hemorrhage, ischemic stroke (thrombotic or embolic), or stroke of unknown causes).^29^

Deaths were identified by reports from next of kin, the US postal service, state vital statistics departments, and systematic searches of the National Death Index. Follow-up for deaths was more than 98%.^30^ Myocardial infarction deaths were confirmed by autopsy or electrocardiographic findings and enzyme changes characteristic of myocardial infarction before death according to medical records. We did not include sudden deaths of unknown cause in our analyses. Participants with confirmed fatal coronary heart disease included those in whom the underlying cause of death was listed as coronary heart disease on the death certificate and evidence indicated a history of coronary heart disease.^30^ ^31^ When medical records were not available but confirmation was provided through interview or letter, participants were considered to have probable cardiovascular disease. For the current analysis, we included participants with confirmed and probable cardiovascular disease. Analyses that included only participants with confirmed cardiovascular disease produced virtually identical results.

#### Assessment of covariates

We used the main biennial follow-up questionnaires to collect and update information on age, ethnicity (assessed once in 1992 in NHS, 1989 in NHS II, 1986 in HPFS), family history of myocardial infarction, body weight, cigarette smoking, physical activity, and multivitamin use. We also gathered information on menopausal status (NHS and NHS II), use of postmenopausal hormones (NHS and NHS II), oral contraceptive use (NHS II only), and history of hypercholesterolemia and hypertension. We considered participants to have hypercholesterolemia or hypertension when they reported these conditions on the biennial questionnaire or when they reported use of lipid or blood pressure lowering drugs. We determined alcohol intake through food frequency questionnaires. Detailed descriptions of the validity and reproducibility of self-reported body weight, physical activity, and alcohol consumption have been published elsewhere.^32^ ^33^ ^34^

#### Statistical methods

We calculated person time from the return of the baseline questionnaire (1980 for NHS, 1991 for NHS II, and 1986 for HPFS) to the diagnosis of cardiovascular disease, death, or the end of follow-up (30 June 2012 for NHS, 30 June 2013 for NHS II, and 31 January 2012 for HPFS), whichever occurred first. We did not censor participants lost to active follow-up because fatal events were included in the outcomes. When we restricted the analyses to non-fatal events and censored participants because of loss to follow-up we had similar results (data not shown).

We computed cumulative averages of dietary variables, including egg intake, to reduce within person variation and to represent long term diet.^21^ For instance, for the 1999-2001 risk set in NHS II, dietary variables in 1991, 1995, and 1999 were averaged to predict subsequent cardiovascular disease risk. Within each cohort, we divided participants into predefined categories of egg intake (less than one egg per month, one to less than four eggs per month, one to less than three eggs per week, three to less than five eggs per week, five to less than seven eggs per week, and at least one egg per day). The group that consumed less than one egg per month served as the reference group. We used Cox proportional hazard models to examine the association between categories of egg intake and cardiovascular disease. In the main analyses, we used whole egg intake as the exposure. Analyses were first conducted within each cohort separately, and then by pooling data from the three cohorts.

The regression model included age in months as the time scale, stratified by calendar time in two year intervals, and allowed for possible interaction between calendar time and age in the baseline hazards to be accounted for non-parametrically (model 1). In the pooled analysis, we also stratified by cohort, which allowed concomitant stratification for sex. In model 2, we additionally adjusted for race or ethnicity (white, other), family history of myocardial infarction (yes, no), baseline history of hypertension (yes, no), and baseline history of hypercholesterolemia (yes, no). Model 2 also included the following time varying covariates updated every two years: body mass index (<21.0, 21.0-22.9, 23.0-24.9, 25.0-26.9, 27.0-29.9, 30.0-34.9, ≥35.0); smoking status (never, former, current); physical activity (<3.0, 3.0-8.9, 9.0-17.9, 18.0-26.9, ≥27.0 metabolic equivalent of task hours per week); alcohol consumption (g/day in fifths); multivitamin use (yes, no); postmenopausal status and postmenopausal hormone use (premenopausal, never, former, current, NHS and NHS II only); and use of oral contraceptives (never, former, current, NHS II only).

In our final model (model 3), we additionally adjusted for total energy intake and consumption of foods associated with egg intake in the US (eg, red meat, bacon, other processed meat, refined grains, potatoes and French fries, fruits, vegetables, full fat milk, fruit juices, sugar-sweetened beverages, and coffee). As with egg intake, we used a cumulative average update for all dietary variables. We tested for a possible linear trend in the hazard ratios across categories of egg consumption by using the median of each category of egg intake as the dose of egg consumption.^35^ Total cardiovascular disease was analyzed separately from coronary heart disease and stroke.

We used statistical models to estimate the effect on risk of total cardiovascular disease of replacing one whole egg per day with one serving of other foods that are common alternatives (unprocessed red meat, processed red meat, poultry, fish, legumes, nuts, refined grains, whole grains, potatoes, low fat cheese, high fat cheese, reduced fat milk, full fat milk, and yogurt; information on yogurt fat content was not collected in our food frequency questionnaires). The covariates in model 3 were used, and eggs and the alternative foods were included as continuous variables in the same multivariable model. We computed the differences between the β coefficients, variance, and the covariance of eggs and the alternative food to estimate hazard ratios and 95% confidence intervals for the replacement effect.^36^ In these analyses, we assumed that total consumption of different foods is constrained to a certain level for each person (the amount of food is held constant); that the association of egg intake with cardiovascular disease risk is independent of the association with the alternative food intake; and that the intake of other foods in the diet remains constant.^37^

We performed stratified analyses defined a priori by updated body mass index, physical activity, self-reported hypertension (including use of antihypertensive drug treatment), self-reported hypercholesterolemia (including use of lipid lowering drug treatment), family history of myocardial infarction, statin use, smoking status, age, Alternative Healthy Eating Index score,^38^ and self-reported prevalent type 2 diabetes. For each of these variables, we tested for potential effect modification by using likelihood ratio tests for interactions.

We performed several sensitivity analyses. We examined the association of egg intake with cardiovascular disease risk by estimating the risk of incident cardiovascular disease across seven categories of egg intake; at least two eggs per day was the highest category of intake. The risk of cardiovascular disease was also estimated for total egg intake (that is, consumption of whole eggs plus eggs in baked goods). Additionally, we modeled egg intake by using baseline diet only and by using the most recent diet. Because diagnosis of an intermediate endpoint of cardiovascular disease could result in changes in diet or in diet reporting, we evaluated associations when diet updating was stopped: after diagnosis of type 2 diabetes, hypertension, hypercholesterolemia, or angina; also after coronary artery bypass graft, or the start of statin treatment.^39^

We repeated the main analysis by replacing foods associated with egg intake (red meat, bacon, other processed meat, refined grains, fruits, vegetables, potatoes and French fries, fruit juices, full fat milk, sugar-sweetened beverages, and coffee) with the Alternative Healthy Eating Index score in the multivariable model. Instead of pooling the data, we used fixed effect meta-analysis to estimate the overall association of egg intake and cardiovascular disease risk among the three cohorts. Finally, we calculated E values to determine the minimum strength of association on the risk ratio scale that an unmeasured confounder would need to have with egg consumption and cardiovascular disease risk to fully shift the observed association toward a significant association.^40^ All P values are two sided and statistical significance was considered at P values less than 0.05. Statistical analyses were performed using SAS version 9.4 (SAS Institute, Cary, NC).

### Systematic review and meta-analysis of egg consumption and incident cardiovascular disease

We conducted a systematic review and updated meta-analysis based on the current study and previous prospective cohort studies that evaluated the association between egg consumption and cardiovascular disease risk in the general population and among people with type 2 diabetes. The report was conducted using the preferred reporting items for systematic reviews and meta-analyses (PRISMA) guidelines.^41^ We registered the protocol on the international prospective register of systematic reviews (PROSPERO CRD42019129650). Supplemental table 1 presents the strategy used to search PubMed, Embase, and Web of Science up to 6 August 2019. We screened the reference lists of selected studies to identify additional relevant studies. Studies were included if they were of prospective design; if they assessed the association between egg consumption and incidence of cardiovascular disease (total cardiovascular disease; total, fatal, and non-fatal coronary heart disease; and total, ischemic, hemorrhagic, fatal and non-fatal stroke); and if they provided risk estimates for three or more levels of egg consumption or a dose-response estimate.

Extracted data included first author name, publication year, cohort name, country where the study was conducted, follow-up duration, number of participants, sex, age range at baseline, method used to assess diet, and method used to identify events. Additionally, we obtained data about cardiovascular disease endpoints, number of events, categories of egg consumption, risk estimates (95% confidence intervals) from the multivariable model, and covariates in the maximally adjusted model. Study authors were contacted by email if information was missing. We used the Newcastle-Ottawa scale to assess the risk of bias in included studies.^42^ Age, sex, body mass index, smoking status, physical activity, alcohol intake, and energy intake were considered primary confounders of the association between egg consumption and cardiovascular disease risk. Hypertension, dyslipidemia, and red meat intake were considered secondary confounders. Two authors (JPDC and SC) independently screened the literature (title and abstract, then full article), extracted the data, and conducted the risk of bias assessment in individual studies. Disagreement and discordance were resolved by consensus between the two authors.

We used 50 g as the standard weight for one egg. Relative risks were used as the common measure of association across studies and hazard ratios were considered equivalent to relative risks. We used the median of each egg intake category if available, or the midpoint between the upper and lower bound, to determine the amount of egg consumption. When the highest category was open (eg, at least one egg per day), we multiplied the lower bound of the highest category by 1.75. One study used the highest category of egg intake as the reference category, and so we back calculated risk estimates and confidence intervals to set the lowest category of intake as the reference group.^43^ In studies without dose-response estimates, we calculated the relative risk for an increase of one egg per day by using the trend for the log relative risk, which accounted for correlated estimates.^35^ ^44^ In studies with only dose-response estimates, we calculated the relative risk for one egg per day and used this estimate in the meta-analysis of high versus low egg consumption. For studies that did not publish person years for each category of egg intake,^6^ ^8^ ^9^ ^45^ ^46^ ^47^ ^48^ we imputed person years based on available data.

We used random effects models to compute the pooled relative risk for cardiovascular disease for the highest category of egg intake compared with the lowest category, and for one egg per day increase. We preferentially used the risk estimate for total cardiovascular disease when available. When studies reported risk estimates for coronary heart disease and stroke, but not for total cardiovascular disease events, risk estimates for coronary heart disease and stroke were pooled by****using fixed effect meta-analysis. We used the pooled estimate in the cardiovascular disease meta-analysis. For stroke, we preferentially used the risk estimate for total stroke when available. When studies reported risk estimates for ischemic stroke and hemorrhagic stroke, but not for total stroke, risk estimates for ischemic stroke and hemorrhagic stroke were pooled using fixed effect meta-analysis. We used the pooled estimate in the stroke meta-analysis. Lastly, we computed the pooled relative risk for the composite of cardiovascular disease, which included only risk estimates for total cardiovascular disease events from studies that reported such estimates.

Heterogeneity was assessed with the I^2^ statistic and interpreted according to the Cochrane Handbook thresholds (0-40%, might not be important; 30-60%, might represent moderate heterogeneity; 50-90%, might represent substantial heterogeneity; 75-100%, considerable heterogeneity).^49^ We conducted an influence analysis by systematically removing each study from the meta-analysis and calculating the relative risk to evaluate if any single study caused the heterogeneity. We also conducted univariate meta-regressions by using study level data to explore potential sources of heterogeneity. Geographical location, sex, follow-up duration, number of participants, number of events, risk of bias, and dietary assessment method were identified a priori as potential sources of heterogeneity. We tested for publication bias by using Begg’s test and Egger’s test, and visual appreciation of a funnel plot. Statistical analyses for the meta-analysis were preformed using Stata version 15.1 (StataCorp, College Station, TX).

### Patient and public involvement

No patients were involved in setting the research question or the outcome measures, nor were they involved in the design and implementation of the study. 

## Results

### Cohort analyses

Over 5 540 314 person years of follow-up (2 406 915 person years in NHS, 2 137 939 person years in NHS II, and 995 460 person years in HPFS), we documented a total of 14 806 participants with cardiovascular disease (7411 in NHS, 1225 in NHS II, and 6170 in HPFS); this total included 9010 participants with coronary heart disease and 5903 participants with stroke. At baseline (1980 in NHS, 1991 in NHS II, and 1986 in HPFS), mean whole egg intake was 0.42 egg per day in NHS, 0.18 egg per day in NHS II, and 0.34 egg per day in HPFS. Egg intake in NHS and HPFS decreased between 1980 and 1994, and then remained stable in later years (supplemental fig 2). In NHS II, mean egg intake was lower than in NHS and HPFS, but remained relatively stable during follow-up. Over the follow-up period, whole egg intake contributed on average to 88%, 74%, and 76% of total egg consumption (consumption of whole eggs plus eggs in baked goods) in NHS, NHS II, and HPFS, respectively.

In 1998 for NHS and HPFS, and 1999 for NHS II (approximately the midpoint of follow-up), participants with a higher egg intake had a higher body mass index, were less physically active in NHS and NHS II, and were more likely to be current smokers in HPFS. These participants were also less likely to be treated with statins or to have a family history of myocardial infarction (supplemental table 2), and they were more likely to have type 2 diabetes. A higher egg intake was associated with higher intakes of calories, unprocessed red meat, bacon, other processed meats, refined grains, potatoes, full fat milk, coffee, and sugar-sweetened beverages. In 1998-99, a total of 2524 of 203 364 participants (1.24%) consumed at least one egg per day in the three cohorts. Of those, 414 of 203 364 (0.20%) consumed at least two eggs per day.

In pooled analyses adjusted only for age (model 1; table 1), participants who consumed at least one egg per day had a non-significant higher hazard ratio for cardiovascular disease of 1.10 (95% confidence interval 0.97 to 1.23) compared with infrequent egg consumers (less than one egg per month). After we accounted for updated lifestyle and dietary characteristics associated with egg intake (model 3; table 1), the association seemed to be reversed, but remained non-significant (pooled hazard ratio 0.93, 95% confidence interval 0.82 to 1.05). When we examined coronary heart disease and stroke separately while accounting for updated lifestyle and diet covariates (model 3; table 1), we found no association with risk among those who consumed at least one egg per day compared with those who consumed less than one egg per month (0.90, 0.77 to 1.05 for coronary heart disease; 0.99, 0.81 to 1.22 for stroke). For total cardiovascular disease, coronary heart disease, and stroke, similar patterns were seen in all three cohorts individually (supplemental tables 3-5); results were similar when we used a fixed effects meta-analysis to pool results (supplemental table 6). We also examined the association between egg intake and cardiovascular disease risk among seven categories of egg intake by including at least two eggs per day as the highest category. No association was found with risk of cardiovascular disease among participants who consumed at least two eggs per day (pooled hazard ratio for at least two eggs per day compared with less than one egg per month 0.91, 95% confidence interval 0.72 to 1.15; supplemental table 7).

**Table 1:** Pooled (three US cohorts) multivariable adjusted hazard ratios (95% confidence intervals) for incident cardiovascular disease according to categories of whole egg consumption

Outcome	Frequency of egg consumption*						P value for trend†	Hazard ratio (95% CI) for 1 egg per day increase
	<1 per month	1 to <4 per month	1 to <3 per week	3 to <5 per week	5 to <7 per week	≥1 per day		
Total cardiovascular disease								
No of events/person years	1058/457 330	3364/1 436 094	6416/2 197 074	3042/1 159 160	533/166 930	393/123 726	—	—
Incidence rate (per 10^5^ person years)	231	234	292	262	319	318	—	—
Model 1	1.00 (reference)	1.00 (0.93 to 1.08)	1.01 (0.95 to 1.08)	1.03 (0.96 to 1.11)	1.18 (1.06 to 1.31)	1.10 (0.97 to 1.23)	0.002	1.09 (1.03 to 1.15)
Model 2	1.00 (reference)	1.01 (0.94 to 1.09)	1.03 (0.97 to 1.11)	1.01 (0.94 to 1.08)	1.12 (1.01 to 1.25)	1.03 (0.91 to 1.16)	0.22	1.04 (0.98 to 1.10)
Model 3	1.00 (reference)	0.98 (0.91 to 1.06)	0.98 (0.91 to 1.05)	0.92 (0.85 to 1.00)	1.01 (0.90 to 1.13)	0.93 (0.82 to 1.05)	0.16	0.98 (0.92 to 1.04)
Coronary heart disease								
No of events/person years	694/457 612	2040/1 437 096	3727/2 199 070	1937/1 159 993	349/167 056	263/123 830	—	—
Incidence rate (per 10^5^ person years)	152	142	169	167	209	212	—	—
Model 1	1.00 (reference)	0.99 (0.91 to 1.08)	1.00 (0.92 to 1.09)	1.04 (0.95 to 1.14)	1.19 (1.05 to 1.36)	1.09 (0.95 to 1.26)	0.003	1.09 (1.01 to 1.16)
Model 2	1.00 (reference)	1.00 (0.91 to 1.09)	1.03 (0.95 to 1.12)	1.02 (0.93 to 1.11)	1.14 (1.00 to 1.30)	1.03 (0.89 to 1.20)	0.13	1.05 (0.98 to 1.12)
Model 3	1.00 (reference)	0.96 (0.88 to 1.05)	0.96 (0.88 to 1.05)	0.91 (0.83 to 1.00)	0.99 (0.86 to 1.14)	0.90 (0.77 to 1.05)	0.22	0.96 (0.89 to 1.04)
Stroke								
No of events/person years	371/457 738	1345/1 437 338	2742/2 199 257	1121/1 160 283	189/167 111	135/123 853	—	—
Incidence rate (per 10^5^ person years)	81	94	125	97	113	109	—	—
Model 1	1.00 (reference)	1.02 (0.90 to 1.14)	1.03 (0.92 to 1.15)	1.01 (0.89 to 1.14)	1.16 (0.97 to 1.38)	1.12 (0.92 to 1.37)	0.16	1.10 (1.00 to 1.21)
Model 2	1.00 (reference)	1.03 (0.91 to 1.15)	1.04 (0.93 to 1.16)	0.98 (0.87 to 1.11)	1.10 (0.92 to 1.31)	1.04 (0.85 to 1.28)	0.88	1.04 (0.95 to 1.15)
Model 3	1.00 (reference)	1.01 (0.90 to 1.14)	1.00 (0.89 to 1.13)	0.94 (0.82 to 1.06)	1.04 (0.86 to 1.25)	0.99 (0.81 to 1.22)	0.53	1.01 (0.91 to 1.12)

* Multivariable adjusted hazard ratios were estimated from Cox proportional hazard models. Model 1: adjusted for age (months), and stratified by calendar time (in two year intervals) and cohort. Model 2: model 1+race (white, other); family history of myocardial infarction (yes, no); baseline hypercholesterolemia (yes, no); baseline hypertension (yes, no); smoking status (never, former, current); body mass index (<21.0, 21.0-22.9, 23.0-24.9, 25.0-26.9, 27.0-29.9, 30.0-34.9, ≥35.0); physical activity (metabolic equivalent of task hours per week: <3.0, 3.0-8.9, 9.0-17.9, 18.0-26.9, ≥27.0); oral contraceptive use (never, former, current, in NHS II only); postmenopausal hormone use (premenopausal, never, former, current, in NHS and NHS II only); alcohol intake (g/day in fifths); and multivitamin use (yes, no). All covariates (except race, family history of myocardial infarction, baseline hypercholesterolemia and hypertension) were updated every two years. Model 3: model 2+updated cumulative average of daily intake of total calories (kcal/day in fifths), full fat milk, bacon, unprocessed red meat, other processed meats, refined grains, fruits, vegetables, potatoes, coffee, fruit juices, and sugar-sweetened beverages (all servings/day in categories).

† P values for trend based on continuous egg variable.

In sensitivity analyses, we found no interaction between egg intake and key variables on cardiovascular disease risk (supplemental table 8): age (<60 *v* ≥60); body mass index (<25 *v* ≥25); physical activity (<15 *v* ≥15 metabolic equivalent of task hours per week); smoking (never *v* ever smoker); hypertension (yes *v* no); hypercholesterolemia (yes *v* no); family history of myocardial infarction (yes *v* no); statin use (yes *v* no); or Alternative Healthy Eating Index score (<median *v* ≥median). We documented a significant interaction between egg consumption and prevalent type 2 diabetes status (P for interaction <0.001). However, egg intake was not associated with cardiovascular disease risk among participants with type 2 diabetes (hazard ratio for at least one egg per day compared with less than one egg per month 1.06, 95% confidence interval 0.81 to 1.39) or among those without type 2 diabetes (0.93, 0.81 to 1.06).

We found no association when we considered total egg intake (consumption of whole eggs plus eggs in baked goods) in the analysis (pooled hazard ratio for one egg per day increase 0.98, 95% confidence interval 0.93 to 1.03; supplemental table 9). When diet was not updated after a diagnosis of hypertension, hypercholesterolemia, type 2 diabetes, or angina, or after coronary artery bypass graft or the start of statin treatment, daily egg consumption was not associated with cardiovascular disease risk (hazard ratio for at least one egg per day compared with less than one egg per month 0.97, 95% confidence interval 0.91 to 1.03). We also found no apparent differences in results when we used a simple update of diet rather than a cumulative average (1.00, 0.90 to 1.10), or when we used baseline diet only (0.98, 0.90 to 1.07). When we adjusted for the Alternative Healthy Eating Index score instead of foods associated with egg intake in the multivariable model, results were similar (pooled hazard ratio for at least one egg per day compared with less than one egg per month 0.96, 95% confidence interval 0.85 to 1.08).

We used the pooled hazard ratio for cardiovascular disease risk for each one egg per day increase (0.98, 95% confidence interval 0.92 to 1.04) for E value calculations. We estimated that an unmeasured confounder associated with egg consumption and cardiovascular disease by a risk ratio of 1.28-fold each could shift the confidence interval to exclude the null toward an inverse association (that is, to bring the upper confidence limit of 1.04 to an upper limit of 0.99), but weaker confounding could not. Conversely, an unmeasured confounder associated with egg consumption and cardiovascular disease by a risk ratio of at least 1.43-fold each could shift the confidence interval to exclude the null toward a positive association (that is, to bring the lower confidence limit of 0.92 to a lower limit of 1.01).

We statistically modeled the replacement of one whole egg per day by one serving per day of another food (supplemental fig 3). We found a higher risk of cardiovascular disease when eggs were replaced with processed red meat (hazard ratio 1.15, 95% confidence interval 1.05 to 1.27), unprocessed red meat (1.10, 1.02 to 1.18), or full fat milk (1.11, 1.03 to 1.20). Statistical model based replacement of one whole egg per day with one daily serving of fish, poultry, legumes, nuts, whole or refined grains, potatoes, reduced fat milk, cheese (low fat or full fat), or yogurt was not associated with cardiovascular disease risk.

### Systematic review and meta-analysis

We screened a total of 763 studies, and 27 studies (28 including the current study) met inclusion criteria (supplemental fig 4).^5^ ^6^ ^8^ ^9^ ^10^ ^11^ ^12^ ^45^ ^46^ ^47^ ^48^ ^50^ ^51^ ^52^ ^53^ ^54^ ^55^ ^56^ ^57^ ^58^ ^59^ ^60^ ^61^ ^62^ ^63^ ^64^ ^65^ Supplemental table 10 presents characteristics of the included studies. Supplemental table 11 shows the list of covariates used in the multivariable model of each study. Fifteen of the 28 studies controlled for all primary confounders and six controlled for primary and secondary confounders. Supplemental table 12 presents the assessment of risk of bias using the Newcastle-Ottawa scale. Twelve studies obtained a score of at least seven and were considered at low risk of bias.

**Fig 1:**
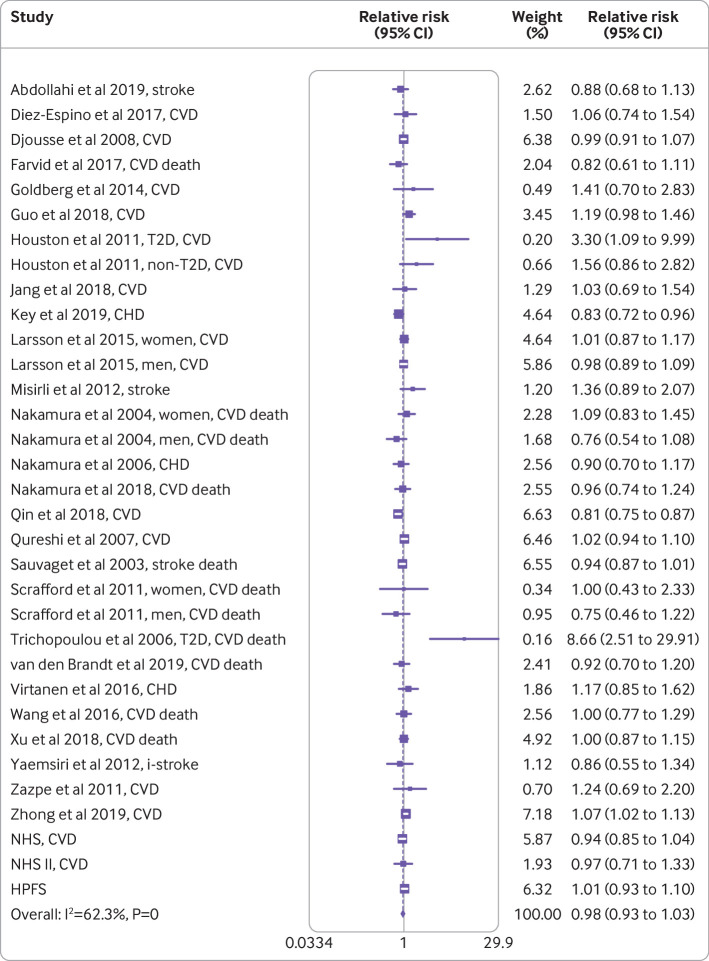
Association of egg consumption with cardiovascular disease risk for one egg per day increase using random effects meta-analysis. Weights of each estimate are represented by size of square. Hollow squares represent individual estimate effects and solid lines represent 95% confidence intervals. Overall effect estimate and 95% confidence interval are represented by diamond and dotted line. I^2^ refers to proportion of heterogeneity among studies. CHD=coronary heart disease; CVD=cardiovascular disease; HPFS=Health Professionals’ Follow-Up Study; i-stroke=ischemic stroke; NHS=Nurses’ Health Study; T2D=type 2 diabetes

The meta-analysis for the association between egg consumption and risk of cardiovascular disease for each one egg per day increase comprised 33 risk estimates, 1 720 108 participants, and 139 195 events (fig 1). The pooled relative risk for cardiovascular disease for one egg per day increase was 0.98 (95% confidence interval 0.93 to 1.03). Similarly, people in the highest category of egg consumption were not at a higher risk of cardiovascular disease compared with people with low egg intake (0.99, 0.93 to 1.06; table 2). Meta-analyses of the association between egg consumption and risk of coronary heart disease, stroke, and composite of cardiovascular disease provided similar results (table 2).

**Table 2:** Meta-analysis of multivariable relative risks of association between egg consumption and cardiovascular disease using random effects models

Outcome	No of risk estimates	No of participants	No of events	Pooled relative risk (95% CI)	I^2 ^(%)
Cardiovascular disease*					
One egg per day increase	33	1 720 108	139 195	0.98 (0.93 to 1.03)	62.3
High *v* low intake	34	1 730 088	139 259	0.99 (0.93 to 1.06)	52.9
Coronary heart disease					
One egg per day increase	21	1 411 261	59 713	0.96 (0.91 to 1.03)	38.2
High *v* low intake	22	1 421 241	59 777	0.97 (0.91 to 1.04)	42.1
Stroke†					
One egg per day increase	22	1 059 315	53 617	0.99 (0.91 to 1.07)	71.5
High *v* low intake	22	1 059 315	53 617	0.96 (0.88 to 1.06)	53.0
Cardiovascular disease composite‡					
One egg per day increase	17	940 464	112 377	1.01 (0.93 to 1.11)	76.6
High *v* low intake	17	940 464	112 377	1.05 (0.94 to 1.17)	67.3
People with type 2 diabetes					
Cardiovascular disease*:					
One egg per day increase	9	28 608	3663	1.25 (0.99 to 1.59)	64.6
High *v* low intake	10	>28 608§	4774	1.40 (1.00 to 1.97)	65.1

* Cardiovascular disease meta-analysis includes risk estimates from all included studies. The risk estimate for total cardiovascular disease events was used when available. For studies that reported risk estimates for coronary heart disease and stroke, but not for total cardiovascular disease events, risk estimates for coronary heart disease and stroke were pooled using fixed effect meta-analysis, and pooled estimates were used in cardiovascular disease meta-analysis.

† For stroke, risk estimate for total stroke was used when available. For studies that reported risk estimates for ischemic stroke and hemorrhagic stroke, but not for total stroke, risk estimates for ischemic stroke and hemorrhagic stroke were pooled using fixed effect meta-analysis, and pooled estimates were used in stroke meta-analysis.

‡ Cardiovascular disease composite meta-analysis includes only risk estimates for total cardiovascular disease events from studies that reported risk estimates for total cardiovascular disease events.

§ In one study, the number of participants with type 2 diabetes was not provided.

We found no evidence of publication bias for the association between egg consumption and cardiovascular disease risk for one egg per day increase (supplemental fig 5), but evidence indicated substantial heterogeneity (I^2^=62.3%). No single study appeared to cause the heterogeneity, although the study by Qin and colleagues^10^ of the China Kadoorie Biobank cohort and the study by Zhong and colleagues^8^ of the Lifetime Risk Pooling Project pulled the association in opposite directions (supplemental fig 6). We found no significant interaction in prespecified subgroup meta-regressions for cardiovascular disease risk for one egg per day increase. However, geographical location (US, Europe, Asia) appeared to be the main source of heterogeneity (P for interaction=0.07). An increase of one egg per day was associated with a lower risk of cardiovascular disease among studies conducted in Asia (relative risk 0.92, 95% confidence interval 0.85 to 0.99), but not among studies conducted in the US (1.01, 0.96 to 1.06) or Europe (1.05, 0.92 to 1.19; table 3). Heterogeneity was minimal among US studies (I^2^=30.8%), moderate among Asian studies (I^2^=44.8%), and substantial among European studies (I^2^=64.7%). In other subgroup analyses, heterogeneity was minimal among studies conducted in men and women separately, among studies with at least 10 years of follow-up, and among studies with low risk of bias (table 3). These different strata all provided similar results and suggested no association between one egg per day increase and risk of cardiovascular disease.

**Table 3:** Prespecified subgroup meta-analyses of cardiovascular disease risk for one egg per day increase using random effects models

Stratification and categories	No of risk estimates	No of participants	No of events	Pooled relative risk (95% CI)	I^2^ (%)	P for interaction
Region						
US	13	503 727	30 796	1.01 (0.96 to 1.06)	30.8	0.07
Europe	10	531 234	18 299	1.05 (0.92 to 1.19)	64.7	—
Asia	10	685 147	90 100	0.92 (0.85 to 0.99)	44.8	—
Sex						
Both	18	1 291 909	109 236	0.99 (0.91 to 1.08)	77.0	0.89
Men	8	116 821	16 342	1.00 (0.94 to 1.06)	21.2	—
Women	7	311 378	13 617	0.97 (0.90 to 1.04)	0.0	—
Follow-up duration						
<10 years	11	624 811	87 654	1.05 (0.87 to 1.27)	70.8	0.43
≥10 years	22	1 095 297	51 541	0.99 (0.95 to 1.03)	35.9	—
No of people						
<10 000	16	67 924	7073	1.05 (0.94 to 1.17)	45.7	0.15
≥10 000	17	1 652 184	132 122	0.96 (0.90 to 1.01)	70.4	—
No of events						
<1000	19	214 735	6655	1.05 (0.95 to 1.17)	41.5	0.13
≥1000	14	1 505 373	132 540	0.95 (0.90 to 1.01)	75.0	—
Risk of bias*						
Low	15	936 243	37 492	0.98 (0.94 to 1.02)	0.0	0.99
High	18	783 865	101 703	0.99 (0.91 to 1.07)	76.1	—
Dietary assessment						
Baseline only	24	916 680	34 844	0.99 (0.93 to 1.05)	52.0	0.56
Repeated measurements	9	803 428	104 351	0.97 (0.88 to 1.06)	70.5	—

* Newcastle-Ottawa scale score: low, ≥7; high, ≤6.

When we restricted the meta-analysis of egg consumption and cardiovascular disease risk to people with type 2 diabetes only (table 2), the pooled relative risk for each egg per day increase was 1.25 (95% confidence interval 0.99 to 1.59) and the pooled relative risk for high versus low intake was 1.40 (1.00 to 1.97). Evidence of considerable heterogeneity existed between studies.

## Discussion

### Principal findings

We found no association between egg consumption and risk of cardiovascular disease in three large US cohorts. Results from the updated meta-analysis lend further support to the overall lack of an association between moderate egg consumption (up to one egg per day) and cardiovascular disease risk. However, evidence of considerable heterogeneity existed between studies (I^2^=62.3%), probably caused by discrepancies in the association between egg consumption and cardiovascular disease risk among studies conducted in the US, Europe, and Asia. No association existed between egg consumption and cardiovascular disease risk among US (minimal heterogeneity, I^2^=30.8%) and European cohorts (substantial heterogeneity, I^2^=64.7%), but an inverse association was found in Asian cohorts (moderate heterogeneity, I^2^=44.8%).^49^ Subgroup analyses revealed additional strata (studies conducted only among men or women, studies with ≥10 years of follow-up, or studies with low risk of bias) with minimal heterogeneity among which no association between egg consumption and cardiovascular disease risk was observed. Finally, when we restricted the meta-analysis to people with type 2 diabetes only, high egg consumption was associated with a higher risk of cardiovascular disease, but considerable heterogeneity between studies was observed concomitantly.

### Strengths, limitations, and interpretation in relation to other studies

We found no association between egg consumption and cardiovascular disease risk in either men or women, which corresponds to previously published analyses of NHS and HPFS.^7^ This lack of an appreciable association is supported by the mean pooled hazard ratios for one egg per day increase for total cardiovascular disease (0.98, 95% confidence interval 0.92 to 1.04), coronary heart disease (0.96, 0.89 to 1.04), and stroke (1.01, 0.91 to 1.12) being close to the null; their 95% confidence intervals being quite narrow and restrained to a level where potential associations would not be clinically meaningful for such an increase in consumption; and E values suggesting the unlikeliness that confounders that could shift the association toward a substantially lower or higher risk were omitted. Overall our cohort analyses are concordant with most analyses conducted in other prospective US cohort studies.^6^ ^12^ ^46^ ^58^ ^65^ However, a recent study conducted in the Lifetime Risk Pooling Project,^8^ which comprises 30 000 people from six prospective US cohorts followed for 17.5 years, reported a positive association between egg consumption and cardiovascular disease risk; this finding reignited the debate on eggs, dietary cholesterol, and cardiovascular health. In that study, each additional 0.5 egg per day increase was associated with a higher risk of coronary heart disease (hazard ratio 1.07, 95% confidence interval 1.01 to 1.12) and stroke (1.10, 1.03 to 1.18).^8^ The observed positive associations at such low intakes could be attributable to the lack of simultaneous control for dietary confounders (such as red meat) and body mass index, which could have led to an overestimate of the association.

Despite recent controversies raised by the Lifetime Risk Pooling Project, no association between egg consumption and risk of cardiovascular disease was found in our meta-analysis of US studies (pooled relative risk for one egg per day increase 1.01, 95% confidence interval 0.96 to 1.06, I^2^=30.8). The pooled relative risk was close to the null, the 95% confidence interval was narrow and restrained to non-clinically meaningful potential associations, and the heterogeneity between US studies was minimal. Therefore, our meta-analysis provides compelling evidence that supports the lack of an appreciable association between egg consumption and risk of cardiovascular disease among US studies.

Major analyses among European and Asian cohorts were also published recently. In the European Prospective Investigation into Cancer and Nutrition cohort, which comprises about 400 000 people from 10 European countries, each additional 20 g of egg per day was associated with a 7% lower risk of coronary heart disease (hazard ratio 0.93, 95% confidence interval 0.88 to 0.99).^11^ However, the inverse association was no longer significant when the first four years of follow-up were censored, which suggests that the results could have been influenced by reverse causation.^11^ Our meta-analysis indicates that egg consumption is not associated with cardiovascular disease risk among Europeans (pooled relative risk for one egg per day increase 1.05, 95% confidence interval 0.92 to 1.19, I^2^=64.7%). However, the 95% confidence interval included a potential moderately higher risk of cardiovascular disease (up to 19% higher risk) and considerable heterogeneity existed between studies, therefore further analyses among European cohorts are required to increase the certainty of the lack of an association.

In the China Kadoorie Biobank study of nearly 0.5 million Chinese adults, higher egg consumption was associated with a lower incidence of cardiovascular disease compared with non-consumers.^10^ When we pooled data from studies conducted in Asia, the China Kadoorie Biobank study had the most weight, and we observed an inverse association between egg intake and cardiovascular disease risk. This result contrasts with data from US and European cohorts, and appeared to have caused the observed heterogeneity in the meta-analysis. However, a combination of factors might explain why a strong inverse association was observed in the China Kadoorie Biobank study. People with the highest egg intake consumed, on average, only 0.76 egg per day.^10^ This is lower than the highest levels of intake in other Asian or Western countries.^66^ When we consider these low levels of intake, it is possible that the association was mainly driven by the egg consumption pattern rather than by egg consumption per se.

In Asian cultures, eggs are typically incorporated into various cuisines, while in Western populations, eggs are typically consumed with red and processed meats and refined grains. Additionally even though analyses of the China Kadoorie Biobank were adjusted for household income and education level, the inverse association could reflect a social gradient. People with higher egg intake were more likely to live in urban areas: 57% of people consuming eggs seven days a week were living in urban areas, whereas only 30% of people who never or rarely consumed eggs were living in urban areas.^10^ Moreover, participants who consumed eggs almost daily and lived in urban areas appeared to have a lower risk of cardiovascular disease compared with participants who consumed eggs almost daily but lived in rural areas.^10^ Thus, data from the China Kadoorie Biobank study are probably affected by residual confounding related to egg consumption patterns and socioeconomic status. Therefore, the inverse association observed in the meta-analysis among Asian cohorts needs to be interpreted cautiously.

The results of our statistical model based replacement analyses suggest that consuming eggs instead of full fat milk, unprocessed red meat, or processed red meat is associated with lower risk of cardiovascular disease. In addition to the estimated beneficial effect on cardiovascular health, replacing red meat with eggs in the diet might also contribute to a more sustainable environment because egg production has a lower environmental impact than meat production.^18^ ^67^ However, when eggs were replaced with fish, poultry, legumes, nuts, whole grains, refined grains, potatoes, reduced fat milk, cheese, or yogurt, we found no significant association with cardiovascular disease.

Results from our updated meta-analysis suggest that higher egg consumption could be associated with a higher risk of cardiovascular disease among people with type 2 diabetes. Insulin resistance is associated with increased endogenous cholesterol synthesis and decreased clearance of cholesterol rich lipoproteins.^68^ Therefore, high dietary cholesterol intake might exacerbate cholesterol homeostasis imbalance and increase cardiovascular disease risk in the long term among people with type 2 diabetes. However, data from short term randomized interventions suggest that higher egg consumption has no deleterious impact on cardiovascular disease risk factors among people with diabetes.^69^ ^70^ Further studies are warranted to understand these discrepancies.

### Strengths and weaknesses of the study

In our cohort analyses, the high rates of follow-up and the large sample size represent major strengths. The increase in total cardiovascular disease events from 1124 in our previous publication on eggs^7^ to 14 806 in our current study allowed for more robust analyses. The repeated measurement of lifestyle variables allowed us to adjust for changes in risk factors over time. Additionally the use of cumulative average updates of dietary variables reduced random measurement error by accounting for within person variations in intake. Finally, the updated meta-analysis provides a comprehensive overview of evidence on the association between eggs and cardiovascular disease in the US and globally.

Our findings also need to be interpreted in the context of several limitations. Our cohorts include health professionals and so the findings might not be generalizable to other populations. However, the high educational status of our study participants is an advantage because high quality and reliable data can be collected and possibilities of confounding by socioeconomic factors, which can be difficult to measure, are reduced. Additionally in our cohorts, people with higher egg intake were generally less healthy in many ways, and we recognize that our results could be affected by unmeasured or residual confounding. However, we were able to account for many dietary and lifestyle covariates, including dietary variables typically associated with egg intake such as red and processed meats. Therefore, as indicated by our E value analyses, it remains unlikely that we omitted confounders that could shift the association toward a significantly lower or higher risk.

Dietary data collection with food frequency questionnaires inevitably leads to some measurement errors. Misclassification because of random measurement error and confounding owing to unmeasured dietary items (eg, egg cooking method) could result in an underestimation of the association between egg intake and cardiovascular disease risk given the prospective nature of the study. However, we used the cumulative average updating method for dietary variables to reduce random errors caused by within person variation and to take into account long term dietary habits. Differential errors in dietary data collection could exist, but they are unlikely because diet was reported long before the diagnosis of the disease. We cannot exclude the fact that advances in diagnostic methods for cardiovascular disease in recent decades could have impacted rates of detection over the course of the follow-up in our cohorts. However, analyses that were stratified by calendar time in two year intervals allowed us to control for this limitation. Finally, the replacement analysis is a statistical modeling strategy that used data across the whole population, without identifying people in the cohort who actually replaced eggs with the replacement foods. Therefore, our results from replacement analysis should be interpreted with caution in the context of statistical modeling.^37^

### Conclusions and policy implications

The results from our cohort study and updated meta-analysis show that moderate egg consumption (up to one egg per day) is not associated with cardiovascular disease risk overall. Findings were consistent across multiple participant and study characteristics except for geographical region. We found that egg consumption was associated with a slightly lower cardiovascular disease risk among Asian cohorts. However, mean egg consumption in the three US cohorts in our study and in cohorts included in the meta-analysis was relatively low. This consumption level should be taken into account when interpreting our results because most participants consumed one to less than five eggs per week, and relatively few participants consumed at least one egg per day.

**What is already known on this topic**

The association between egg consumption and cardiovascular disease risk has been a topic of intense debate during the past decade
Findings from previous studies on egg consumption and risk of cardiovascular disease have been inconclusive

**What this study adds**

Results from this cohort study and updated meta-analysis show that moderate egg consumption (up to one egg per day) is not associated with cardiovascular disease risk overall
Results were similar for coronary heart disease and stroke
Egg consumption seems to be associated with a slightly lower cardiovascular disease risk among Asian cohorts

## Web extra

Extra material supplied by authors

**Web appendix:** Supplemental material

## Notes

Contributors: JPDC and SC contributed equally to the manuscript. JPDC and SNB have full access to all of the data in the study and take responsibility for the integrity of the data and the accuracy of the data analysis. SNB, ALS, YL, JPDC, and FBH were involved in the study conception and design. JPDC and SC conducted the systematic review and meta-analysis. JPDC, YL, SC, SNB, and ALS analyzed and interpreted the data. SNB, YL, BR, and FBH provided statistical expertise. JPDC and SNB drafted the manuscript. All the authors participated in the interpretation of the results and critical revision of the manuscript. SNB and FBH are the guarantors. The corresponding author attests that all listed authors meet authorship criteria and that no others meeting the criteria have been omitted.

Ethical approval: The institutional review boards of Brigham and Women’s Hospital and the Harvard T H Chan School of Public Health approved this study. Return of the mailed questionnaire was considered to imply informed consent. Protocol number: 2009-P-002375.

Data sharing: Data described in the manuscript, code book, and analytic code will not be made publicly available. Further information including the procedures for obtaining and accessing data from the Nurses’ Health Studies and Health Professionals’ Follow-up Study is described at https://www.nurseshealthstudy.org/researchers (email: nhsaccess@channing.harvard.edu) and https://sites.sph.harvard.edu/hpfs/for-collaborators

The manuscript’s guarantors affirm that the manuscript is an honest, accurate, and transparent account of the study being reported; that no important aspects of the study have been omitted; and that any discrepancies from the study as planned (and, if relevant, registered) have been explained.

Dissemination to participants and related patient and public communities: No patients were involved in setting the research question or the outcome measures, nor were they involved in the design and implementation of the study. We plan to disseminate these findings to participants in our annual newsletter and to the general public in a press release.
