# Supplementary material for: Egg consumption and risk of cardiovascular disease: three large prospective US cohort studies, systematic review, and updated meta-analysis
Source: BMJ. 2020 Mar 4;368:m513. doi: 10.1136/bmj.m513 (PMC7190072; doi:10.1136/bmj.m513)
Supplement: Supplementary file 1 — Web appendix: Supplemental material [file droj050974.ww.pdf]

Supplemental Material

Supplemental Table 1: Systematic review search strategy. 2

Supplemental Table 2: Age and age-standardized characteristics of participants in Nurses' Health Study, Nurses' Health Study II and Health Professionals Follow-Up Study in 1998/1999. 3

‡Includes incident cases until the return of the 1998 questionnaire (1999 in NHS II). 5

Supplemental Table 3: Multivariable adjusted hazard ratios (95% confidence intervals) for incident cardiovascular disease according to categories of whole egg consumption. 6

Supplemental Table 4: Multivariable adjusted hazard ratios (95% confidence intervals) for incident coronary heart disease according to categories of whole egg consumption. 7

Supplemental Table 5: Multivariable adjusted hazard ratios (95% confidence intervals) for incident stroke according to categories of whole egg consumption. 8

Supplemental Table 6: Multivariable adjusted hazard ratios (95% confidence intervals) for incident cardiovascular disease according to categories of whole egg consumption: pooled results from the Nurses' Health Study, the Nurses' Health Study II, and the Health Professional's Follow-up Study using fixed-effects meta-analysis. 9

Supplemental Table 7: Hazard ratios (95% confidence intervals) of incident cardiovascular disease according to seven categories of egg consumption. 10

Supplemental Table 8: Multivariable adjusted hazard ratios (95% confidence intervals) for incident cardiovascular disease according to categories of whole egg consumption stratified by key variables. 11

Supplemental Table 9: Hazard ratios (95% confidence intervals) of incident cardiovascular disease according to categories of total egg consumption, including eggs in mixed foods. 13

Supplemental Table 10: Characteristics of studies included in the meta-analysis. 14

Supplemental Table 11: List of confounders among studies included in the meta-analysis. 25

Supplemental Table 12: Assessment of risk of bias with the Newcastle-Ottawa Scale.* 28

Supplemental Figure 1: Flow chart of participants. 29

Supplemental Figure 2: Trends in mean egg consumption in the Nurses' Health Study, Nurses' Health Study II, and Health Professionals Follow-Up Study over time. 30

Supplemental Figure 3: Statistical model-based hazard ratios and 95% confidence intervals for incident cardiovascular disease associated with replacement of one egg per day with one serving per day of other foods. 31

Supplemental Figure 4: Flow chart of study selection. 32

Supplemental Figure 5: Funnel plot for assessment of publication bias for the association between egg consumption and risk of cardiovascular disease. 33

Supplemental Figure 6: Forest plot of influence analysis for the association between egg consumption and risk of cardiovascular disease. 34

Supplemental Table 1: Systematic review search strategy.

Database	Step	Terms	Results*
Pubmed	1	"Egg" OR "Eggs"	126,331
	2	"Cardiovascular Diseases" [Mesh] OR "Stroke" [Mesh] OR Cardiovascular OR Coronary Heart Disease OR Myocardial Infarction OR CHD OR CVD OR Ischemic Heart Disease OR Ischaemic Heart Disease OR Ischaemic stroke OR Ischemic Stroke OR Haemorrhagic Stroke OR Hemorrhagic Stroke	3,213,209
	3	Prospective OR Cohort OR Longitudinal OR Follow-up OR Case-cohort OR Nested Case-control	2,769,226
	4	human	18,572,110
	5	#1 AND #2 AND #3 AND #4	**216**
Web of Science	1	(TS=(((Egg) AND (Cardiovascular or Stroke or Coronary Heart Disease or Myocardial Infarction or CHD or CVD or Ischemic Heart Disease or Ischaemic Heart Disease)) AND (Prospective or Cohort or Longitudinal or Follow-up or Case-cohort or Nested Case-control))) AND DOCUMENT TYPES: (Article)	**370**
Embase	1	('egg'/exp OR egg OR 'eggs'/exp OR eggs OR egg.ab.) AND [embase]/lim	93,138
	2	('cardiovascular disease'/exp OR cardiovascular.ab. OR 'ischemic heart disease.ab.' OR 'coronary artery disease.ab.' OR 'cerebrovascular accident' OR 'stroke.ab.' OR 'chd.ab.' OR 'cvd.ab.' OR 'coronary heart disease.ab.' OR 'myocardil infarction' OR 'ischaemic heart disease.ab.' OR 'ischaemic stroke.ab.' OR 'ischemic stroke.ab.' OR 'haemorrhagic stroke.ab.' OR 'hemorrhagic stroke.ab.') AND [embase]/lim	3,468,794
	3	('prospective study' OR 'cohort study' OR 'case control study' OR 'longitudinal study' OR 'follow up' OR 'case-cohort' OR 'case-control') AND [embase]/lim	2,281,202
	4	'human' AND [embase]/lim	15,638,594
	5	(#1 AND #2 AND #3 AND #4)	**375**

* Search date: 2019-08-06.

Supplemental Table 2: Age and age-standardized characteristics of participants in Nurses' Health Study, Nurses' Health Study II and Health Professionals Follow-Up Study in 1998/1999.

	Frequency of egg consumption					
	**<1/month**	**1 to <4/month**	**1 to <3/week**	**3 to <5/week**	**5 to <7/week**	**≥1/day**
**Nurses' Health Study (1998)**						
Participants, n	2,700	15,811	41,313	13,350	1,901	1,080
Age, years	64.9 (7.1)	64.0 (7.2)	63.6 (7.1)	63.4 (7.1)	63.1 (7.0)	63.5 (6.9)
Body mass index, kg/m^2	25.5 (5.0)	25.8 (4.9)	26.7 (5.2)	27.6 (5.7)	27.7 (6.3)	28.1 (6.3)
Physical activity, MET-hours/week	19.9 (30.3)	17.5 (21.7)	17.0 (20.6)	16.5 (21.2)	16.4 (21.7)	18.1 (27.2)
White race, %	97	97	98	97	97	97
Smoking Status						
Never smoker, %	41	44	46	49	49	49
Past smoker, %	45	45	44	40	38	39
Current smoker, %	14	11	10	11	12	12
Postmenopausal, %	96	96	96	95	95	93
Current menopausal hormone use, %	40	44	46	41	39	35
Family history of diabetes, %	27	28	28	27	27	24
Family history of myocardial infarction, %	19	20	19	17	18	18
Hypertension[†], %	48	48	50	49	50	46
Hypercholesterolemia[†], %	56	58	56	49	48	45
Type 2 diabetes[‡], %	4	5	6	8	9	10
Current multivitamin use, %	55	58	61	58	57	54
Current statin use, %	19	18	16	10	8	9
Dietary intake						
Total Energy, kcal/day	1,489 (410)	1,543 (387)	1,718 (398)	1,864 (450)	1,901 (479)	1,940 (519)
Whole egg consumption, unit/day	0.01 (0.01)	0.09 (0.03)	0.27 (0.08)	0.50 (0.08)	0.81 (0.07)	1.38 (0.55)
Bacon, servings/day	0.04 (0.07)	0.05 (0.07)	0.08 (0.09)	0.13 (0.13)	0.18 (0.22)	0.20 (0.33)
Unprocessed red meat, servings/day	0.72 (0.49)	0.74 (0.41)	0.81 (0.41)	0.97 (0.49)	1.05 (0.63)	1.07 (0.71)
Other processed meat, servings/day	0.13 (0.17)	0.15 (0.16)	0.19 (0.17)	0.24 (0.22)	0.25 (0.25)	0.27 (0.32)
Refined grains, servings/day	1.08 (0.75)	1.11 (0.72)	1.26 (0.76)	1.43 (0.90)	1.46 (1.08)	1.52 (1.22)
Potatoes, servings/day	0.43 (0.29)	0.43 (0.27)	0.48 (0.27)	0.52 (0.30)	0.53 (0.38)	0.51 (0.38)
Full-fat milk, servings/day	0.11 (0.31)	0.12 (0.29)	0.13 (0.30)	0.19 (0.41)	0.24 (0.47)	0.30 (0.58)
Coffee, servings/day	2.10 (1.60)	2.12 (1.50)	2.22 (1.48)	2.34 (1.59)	2.44 (1.73)	2.35 (1.78)
Juice, servings/day	0.63 (0.58)	0.65 (0.55)	0.73 (0.55)	0.79 (0.60)	0.77 (0.61)	0.76 (0.67)
Sugar sweetened beverages, servings/day	0.32 (0.56)	0.32 (0.51)	0.30 (0.46)	0.34 (0.51)	0.37 (0.63)	0.37 (0.65)
Fruits, servings/day	1.72 (1.20)	1.69 (1.01)	1.85 (0.97)	1.96 (1.08)	1.98 (1.18)	2.04 (1.40)
Vegetables, servings/day	3.18 (1.69)	3.12 (1.51)	3.40 (1.50)	3.43 (1.63)	3.38 (1.80)	3.27 (1.91)
Protein, %	18.3 (3.1)	18.1 (2.7)	18.4 (2.5)	18.5 (2.7)	18.8 (2.9)	19.3 (3.4)
Carbohydrates, %	49.3 (8.3)	48.8 (7.1)	47.8 (6.3)	45.5 (6.6)	43.4 (7.4)	41.7 (8.5)
Total fat, %	31.5 (6.3)	32.2 (5.2)	33.0 (4.7)	35.0 (5.0)	36.6 (5.7)	37.7 (6.4)
Saturated fat, %	11.4 (3.0)	11.6 (2.4)	11.8 (2.1)	12.8 (2.4)	13.5 (2.8)	14.0 (3.1)
Trans fat, g/day	2.78 (1.27)	2.94 (1.17)	3.34 (1.22)	3.82 (1.41)	3.96 (1.60)	4.00 (1.76)
Cholesterol, mg/day	173 (57)	199 (52)	262 (59)	342 (66)	433 (77)	581 (176)

Alcohol, g/day	5.4 (8.8)	5.6 (8.7)	5.8 (8.5)	6.2 (9.3)	6.1 (9.4)	6.2 (11.1)
**Nurses' Health Study II (1999)**						
Participants, n	9,479	36,697	33,243	9,002	815	443
Age, years	44.3 (4.7)	44.0 (4.7)	44.3 (4.6)	44.6 (4.6)	45.0 (4.5)	45.3 (4.6)
Body mass index, kg/m^2	25.5 (5.6)	26.2 (5.9)	26.8 (6.3)	27.5 (6.8)	28.0 (7.0)	29.0 (8.3)
Physical activity, MET-hours/week	22.8 (27.5)	18.8 (23.5)	17.8 (21.9)	17.5 (22.0)	17.4 (26.0)	19.0 (28.1)
White race, %	96	96	97	96	95	95
Smoking Status						
Never smoker, %	65	66	66	67	64	65
Past smoker, %	26	25	25	23	27	26
Current smoker, %	9	9	9	10	9	8
Postmenopausal, %	18	18	19	17	18	18
Current menopausal hormone use, %	12	12	12	11	12	10
Current oral contraceptive use, %	8	8	8	7	7	8
Family history of diabetes, %	31	33	34	33	33	35
Family history of myocardial infarction, %	34	34	34	32	33	31
Hypertension[†], %	14	15	16	17	18	22
Hypercholesterolemia[†], %	25	24	24	23	23	25
Type 2 diabetes[‡], %	1	1	2	2	3	2
Current multivitamin use, %	55	55	57	56	60	62
Current statin use, %	4	3	3	2	3	2
Dietary intake						
Total Energy, kcal/day	1,567 (451)	1,698 (447)	1,905 (466)	2,110 (498)	2,067 (543)	2,149 (527)
Whole egg consumption, unit/day	0.01 (0.01)	0.08 (0.02)	0.23 (0.07)	0.46 (0.07)	0.83 (0.07)	1.32 (0.48)
Bacon, servings/day	0.02 (0.05)	0.04 (0.05)	0.07 (0.09)	0.11 (0.14)	0.18 (0.26)	0.20 (0.28)
Unprocessed red meat, servings/day	0.50 (0.40)	0.64 (0.40)	0.76 (0.44)	0.87 (0.51)	0.87 (0.55)	0.91 (0.65)
Other processed meat, servings/day	0.09 (0.14)	0.14 (0.15)	0.18 (0.17)	0.23 (0.22)	0.23 (0.25)	0.26 (0.34)
Refined grains, servings/day	1.37 (0.83)	1.43 (0.79)	1.62 (0.86)	1.82 (0.99)	1.68 (1.05)	1.93 (1.36)
Potatoes, servings/day	0.43 (0.31)	0.49 (0.30)	0.55 (0.32)	0.62 (0.36)	0.59 (0.47)	0.59 (0.41)
Full-fat milk, servings/day	0.03 (0.17)	0.05 (0.20)	0.06 (0.24)	0.10 (0.32)	0.10 (0.30)	0.13 (0.42)
Coffee, servings/day	1.44 (1.51)	1.50 (1.48)	1.58 (1.49)	1.63 (1.58)	1.68 (1.65)	1.53 (1.63)
Juice, servings/day	0.56 (0.72)	0.59 (0.62)	0.68 (0.66)	0.78 (0.74)	0.68 (0.73)	0.70 (0.71)
Sugar sweetened beverages, servings/day	0.41 (0.74)	0.47 (0.73)	0.50 (0.75)	0.58 (0.83)	0.49 (0.78)	0.52 (1.04)
Fruits, servings/day	1.23 (0.98)	1.17 (0.82)	1.26 (0.81)	1.32 (0.88)	1.34 (1.00)	1.32 (0.96)
Vegetables, servings/day	3.40 (2.16)	3.27 (1.82)	3.59 (1.86)	3.80 (1.98)	3.97 (2.17)	4.01 (2.29)
Protein, %	18.8 (3.3)	18.8 (2.8)	18.8 (2.7)	18.7 (2.8)	19.3 (3.1)	19.7 (3.6)
Carbohydrates, %	53.6 (7.9)	51.5 (6.6)	50.4 (6.3)	49.3 (6.4)	46.8 (7.4)	45.6 (8.3)
Total fat, %	28.3 (6.0)	30.2 (5.0)	31.4 (4.8)	32.6 (4.8)	34.5 (5.5)	35.5 (6.3)
Saturated fat, %	9.7 (2.51)	10.5 (2.1)	10.9 (2.1)	11.4 (2.1)	12.1 (2.4)	12.3 (2.6)
Trans fat, g/day	2.50 (1.25)	2.94 (1.23)	3.37 (1.31)	3.84 (1.47)	3.72 (1.63)	3.79 (1.57)
Cholesterol, mg/day	163 (62)	200 (59)	255 (65)	329 (72)	406 (77)	513 (137)
Alcohol, g/day	3.1 (5.4)	3.3 (5.6)	3.6 (6.0)	3.7 (6.5)	3.4 (6.7)	2.8 (5.8)
**Health Professionals' Follow-up Study (1998)**						
Participants, n	4,814	10,302	13,972	6,380	1,061	1,001

Age, years	63.8 (8.9)	63.6 (9.0)	64.4 (9.2)	65.3 (9.2)	64.9 (9.1)	65.5 (9.1)
Body mass index, kg/m^2	25.4 (3.6)	26.0 (3.5)	26.3 (3.6)	26.7 (4.4)	26.6 (3.8)	26.5 (4.1)
Physical activity, MET-hours/week	37.2 (41.3)	35.3 (41.1)	33.0 (37.8)	33.1 (39.7)	33.5 (38.2)	37.6 (46.4)
White race, %	94	95	95	95	95	95
Smoking Status						
Never smoker, %	55	52	49	47	45	46
Past smoker, %	42	44	44	44	43	42
Current smoker, %	3	5	6	9	12	12
Family history of diabetes, %	15	14	14	13	14	10
Family history of myocardial infarction, %	38	33	30	29	27	25
Hypertension†, %	42	42	42	40	37	34
Hypercholesterolemia†, %	54	50	46	37	35	31
Type 2 diabetes‡, %	3	4	5	6	7	8
Current multivitamin use, %	63	61	60	61	62	62
Current statin use, %	22	17	13	7	6	5
Dietary intake						
Total Energy, kcal/day	1,778 (491)	1,856 (488)	2,013 (515)	2,181 (576)	2,268 (573)	2,367 (592)
Whole egg consumption, unit/day	0.01 (0.01)	0.08 (0.03)	0.25 (0.08)	0.48 (0.08)	0.82 (0.07)	1.56 (0.56)
Bacon, servings/day	0.02 (0.06)	0.05 (0.08)	0.09 (0.12)	0.16 (0.18)	0.25 (0.26)	0.34 (0.41)
Unprocessed red meat, servings/day	0.47 (0.46)	0.64 (0.44)	0.78 (0.47)	0.93 (0.54)	1.01 (0.58)	1.17 (0.72)
Other processed meat, servings/day	0.10 (0.19)	0.16 (0.19)	0.23 (0.23)	0.28 (0.28)	0.31 (0.31)	0.34 (0.39)
Refined grains, servings/day	1.18 (0.83)	1.18 (0.80)	1.30 (0.84)	1.43 (0.97)	1.55 (1.04)	1.57 (1.21)
Potatoes, servings/day	0.46 (0.34)	0.50 (0.32)	0.56 (0.33)	0.62 (0.37)	0.66 (0.40)	0.69 (0.41)
Full-fat milk, servings/day	0.03 (0.17)	0.06 (0.25)	0.10 (0.30)	0.16 (0.40)	0.23 (0.52)	0.29 (0.59)
Coffee, servings/day	1.49 (1.46)	1.74 (1.51)	1.92 (1.54)	2.10 (1.61)	2.14 (1.59)	2.36 (1.87)
Juice, servings/day	0.83 (0.78)	0.78 (0.67)	0.80 (0.65)	0.82 (0.76)	0.79 (0.69)	0.80 (0.75)
Sugar sweetened beverages, servings/day	0.28 (0.49)	0.33 (0.50)	0.36 (0.51)	0.41 (0.56)	0.42 (0.56)	0.40 (0.56)
Fruits, servings/day	1.95 (1.50)	1.70 (1.20)	1.62 (1.06)	1.59 (1.08)	1.62 (1.15)	1.53 (1.12)
Vegetables, servings/day	3.99 (2.37)	3.67 (1.89)	3.68 (1.83)	3.75 (1.85)	3.87 (1.98)	3.81 (1.99)
Protein, %	18.4 (3.1)	18.1 (2.7)	18.0 (2.6)	18.0 (2.7)	18.3 (2.6)	18.7 (2.7)
Carbohydrates, %	53.8 (8.5)	50.6 (7.4)	48.6 (6.7)	46.6 (6.7)	45.2 (6.4)	43.2 (7.2)
Total fat, %	26.8 (6.1)	29.7 (5.2)	31.4 (4.9)	33.1 (4.8)	34.4 (4.8)	35.9 (5.3)
Saturated fat, %	8.4 (2.5)	9.7 (2.2)	10.5 (2.1)	11.3 (2.2)	11.8 (2.2)	12.5 (2.4)
Trans fat, g/day	2.32 (1.28)	2.84 (1.32)	3.32 (1.45)	3.70 (1.66)	3.86 (1.71)	3.99 (1.74)
Cholesterol, mg/day	181 (67)	217 (65)	273 (73)	349 (83)	438 (85)	604 (156)
Alcohol, g/day	9.0 (12.6)	10.1 (12.6)	11.4 (13.3)	12.4 (14.5)	11.8 (13.9)	12.0 (15.8)

* All variables except age are age-standardized. Values are mean (SD) or percentage.

†Includes prevalent cases before baseline and incident cases until the return of the 1998 questionnaire (1999 in NHS II).

‡Includes incident cases until the return of the 1998 questionnaire (1999 in NHS II).

Supplemental Table 3: Multivariable adjusted hazard ratios (95% confidence intervals) for incident cardiovascular disease according to categories of whole egg consumption.

	Frequency of Egg Consumption*						*P* value for trend†	HR (95% CI) per 1 egg per day increase
	<1/month	1 to <4/month	1 to <3/week	3 to <5/week	5 to <7/week	≥1/day		
**Nurses' Health Study**								
Cases/Person years	291/83 722	1 494/404 893	3 701/1 093 801	1 534/646 761	230/103 726	161/74 012		
Model 1	1.00 (Ref.)	0.96 (0.85, 1.09)	0.91 (0.81, 1.03)	0.94 (0.83, 1.07)	0.98 (0.82, 1.17)	1.06 (0.87, 1.29)	0.59	1.06 (0.96, 1.16)
Model 2	1.00 (Ref.)	1.02 (0.90, 1.16)	1.00 (0.88, 1.12)	0.98 (0.86, 1.11)	0.97 (0.82, 1.16)	1.02 (0.84, 1.24)	0.50	1.01 (0.92, 1.11)
Model 3	1.00 (Ref.)	1.01 (0.89, 1.14)	0.96 (0.84, 1.08)	0.91 (0.79, 1.03)	0.90 (0.75, 1.07)	0.93 (0.77, 1.14)	0.03	0.94 (0.85, 1.04)
**Nurses' Health Study II**								
Cases/Person years	130/259 156	414/783 358	520/771 069	126/285 402	28/25 668	7/13 286		
Model 1	1.00 (Ref.)	0.91 (0.75, 1.11)	1.08 (0.89, 1.31)	0.93 (0.73, 1.19)	1.84 (1.22, 2.77)	0.85 (0.39, 1.82)	0.08	1.30 (0.99, 1.70)
Model 2	1.00 (Ref.)	0.89 (0.73, 1.08)	1.03 (0.84, 1.25)	0.85 (0.66, 1.09)	1.54 (1.02, 2.33)	0.74 (0.35, 1.59)	0.47	1.13 (0.85, 1.50)
Model 3	1.00 (Ref.)	0.84 (0.69, 1.03)	0.95 (0.77, 1.17)	0.76 (0.59, 0.99)	1.32 (0.86, 2.02)	0.61 (0.28, 1.31)	0.76	0.97 (0.71, 1.33)
**Health Professionals' Follow-up Study**								
Cases/Person years	637/114 452	1 456/247 843	2 195/332 204	1 382/226 997	275/37 536	225/36 428		
Model 1	1.00 (Ref.)	1.02 (0.93, 1.12)	1.07 (0.98, 1.17)	1.09 (0.99, 1.20)	1.29 (1.12, 1.49)	1.11 (0.95, 1.29)	0.003	1.10 (1.02, 1.18)
Model 2	1.00 (Ref.)	1.01 (0.92, 1.11)	1.05 (0.96, 1.16)	1.04 (0.95, 1.15)	1.23 (1.06, 1.42)	1.05 (0.90, 1.22)	0.07	1.06 (0.98, 1.14)
Model 3	1.00 (Ref.)	0.99 (0.90, 1.09)	1.01 (0.92, 1.11)	0.98 (0.88, 1.09)	1.13 (0.97, 1.32)	0.97 (0.82, 1.14)	0.80	1.01 (0.93, 1.10)
**Pooled Results**								
Cases/Person years	1 058/457 330	3 364/1 436 094	6 416/2 197 074	3 042/1 159 160	533/166 930	393/123 726		
Model 1	1.00 (Ref.)	1.00 (0.93, 1.08)	1.01 (0.95, 1.08)	1.03 (0.96, 1.11)	1.18 (1.06, 1.31)	1.10 (0.97, 1.23)	0.002	1.09 (1.03, 1.15)
Model 2	1.00 (Ref.)	1.01 (0.94, 1.09)	1.03 (0.97, 1.11)	1.01 (0.94, 1.08)	1.12 (1.01, 1.25)	1.03 (0.91, 1.16)	0.22	1.04 (0.98, 1.10)
Model 3	1.00 (Ref.)	0.98 (0.91, 1.06)	0.98 (0.91, 1.05)	0.92 (0.85, 1.00)	1.01 (0.90, 1.13)	0.93 (0.82, 1.05)	0.16	0.98 (0.92, 1.04)

*Multivariable adjusted hazard ratios (HR) were estimated from Cox proportional hazards models.

Model 1: adjusted for age (months), and stratified by calendar time (in 2-year intervals) and cohort.

Model 2: model 1 + race (Caucasian, other), family history of myocardial infarction (yes, no), baseline hypercholesterolemia (yes, no), baseline hypertension (yes, no), smoking status (never, former, current), body mass index (kg/m^2: categorical <21.0, 21.0-22.9, 23.0-24.9, 25.0-26.9, 27.0-29.9, 30.0-34.9, ≥35.0), physical activity (metabolic equivalent of task-hours/week: categorical <3.0, 3.0-8.9, 9.0-17.9, 18.0-26.9, ≥27.0), oral contraceptive use (never, former, current, in NHS II only), postmenopausal hormone use (premenopausal, never, former, current, in NHS and NHS II), alcohol intake (g/d in quintiles), and multivitamin use (yes, no). All covariates (except race, family history of myocardial infarction, baseline hypercholesterolemia and hypertension) were updated every 2 years.

Model 3: model 2 + updated cumulative average of daily intake of total calories (kcals/d in quintiles), full-fat milk (servings/d in categories), bacon (servings/d in categories), red meat (servings/d in categories), other processed meats (servings/d in categories), refined grains (servings/d in categories), fruits (servings/d in categories), vegetables (servings/d in categories), potatoes (servings/d in categories), coffee (servings/d in categories), fruit juices (servings/d in categories), and sugar-sweetened beverages (servings/d in categories).

†*P* values for trend based on continuous egg variable.

Supplemental Table 4: Multivariable adjusted hazard ratios (95% confidence intervals) for incident coronary heart disease according to categories of whole egg consumption.

	**Frequency of Egg Consumption***						***P* value for trend**†	**HR (95% CI) per 1 egg per day increase**
	**<1/month**	**1 to <4/month**	**1 to <3/week**	**3 to <5/week**	**5 to <7/week**	**≥1/day**		
				**Nurses' Health Study**				
Cases/Person years	164/83 803	763/405 392	1 881/1 095 105	858/647 254	137/103 790	93/74 064		
Model 1	1.00 (Ref.)	0.90 (0.76, 1.06)	0.85 (0.72, 0.99)	0.90 (0.76, 1.06)	0.99 (0.79, 1.24)	1.00 (0.77, 1.29)	0.38	1.06 (0.94, 1.20)
Model 2	1.00 (Ref.)	0.96 (0.81, 1.14)	0.95 (0.80, 1.11)	0.95 (0.80, 1.13)	1.00 (0.79, 1.25)	0.98 (0.75, 1.26)	0.93	1.02 (0.90, 1.16)
Model 3	1.00 (Ref.)	0.94 (0.79, 1.12)	0.89 (0.76, 1.05)	0.85 (0.71, 1.01)	0.88 (0.69, 1.11)	0.85 (0.65, 1.11)	0.10	0.92 (0.80, 1.05)
				**Nurses' Health Study II**				
Cases/Person years	72/259 214	221/783 534	282/771 293	57/285 457	18/25 676	3/13 288		
Model 1	1.00 (Ref.)	0.86 (0.66, 1.13)	1.03 (0.79, 1.33)	0.76 (0.53, 1.07)	2.05 (1.22, 3.45)	0.61 (0.19, 1.96)	0.45	1.28 (0.89, 1.84)
Model 2	1.00 (Ref.)	0.82 (0.63, 1.07)	0.96 (0.73, 1.25)	0.66 (0.47, 0.94)	1.62 (0.96, 2.74)	0.55 (0.17, 1.76)	0.91	1.10 (0.75, 1.62)
Model 3	1.00 (Ref.)	0.77 (0.59, 1.01)	0.86 (0.65, 1.14)	0.56 (0.39, 0.81)	1.31 (0.75, 2.27)	0.43 (0.13, 1.38)	0.28	0.91 (0.59, 1.42)
				**Health Professionals' Follow-up Study**				
Cases/Person years	458/114 595	1 056/248 170	1 564/332 672	1 022/227 282	194/37 590	167/36 478		
Model 1	1.00 (Ref.)	1.04 (0.93, 1.16)	1.08 (0.97, 1.20)	1.12 (1.00, 1.26)	1.26 (1.06, 1.49)	1.14 (0.95, 1.37)	0.008	1.09 (1.00, 1.18)
Model 2	1.00 (Ref.)	1.03 (0.92, 1.15)	1.07 (0.96, 1.19)	1.09 (0.97, 1.22)	1.20 (1.01, 1.43)	1.09 (0.90, 1.30)	0.07	1.06 (0.97, 1.15)
Model 3	1.00 (Ref.)	1.00 (0.89, 1.12)	1.02 (0.91, 1.14)	1.01 (0.89, 1.14)	1.09 (0.91, 1.31)	0.99 (0.82, 1.20)	0.80	1.00 (0.91, 1.10)
				**Pooled Results**				
Cases/Person years	694/457 612	2 040/1 437 096	3 727/2 199 070	1 937/1 159 993	349/167 056	263/123 830		
Model 1	1.00 (Ref.)	0.99 (0.91, 1.08)	1.00 (0.92, 1.09)	1.04 (0.95, 1.14)	1.19 (1.05, 1.36)	1.09 (0.95, 1.26)	0.003	1.09 (1.01, 1.16)
Model 2	1.00 (Ref.)	1.00 (0.91, 1.09)	1.03 (0.95, 1.12)	1.02 (0.93, 1.11)	1.14 (1.00, 1.30)	1.03 (0.89, 1.20)	0.13	1.05 (0.98, 1.12)
Model 3	1.00 (Ref.)	0.96 (0.88, 1.05)	0.96 (0.88, 1.05)	0.91 (0.83, 1.00)	0.99 (0.86, 1.14)	0.90 (0.77, 1.05)	0.22	0.96 (0.89, 1.04)

*Multivariable adjusted hazard ratios (HR) were estimated from Cox proportional hazards models.

Model 1: adjusted for age (months), and stratified by calendar time (in 2-year intervals) and cohort.

Model 2: model 1 + race (Caucasian, other), family history of myocardial infarction (yes, no), baseline hypercholesterolemia (yes, no), baseline hypertension (yes, no), smoking status (never, former, current), body mass index (kg/m^2: categorical <21.0, 21.0-22.9, 23.0-24.9, 25.0-26.9, 27.0-29.9, 30.0-34.9, ≥35.0), physical activity (metabolic equivalent of task-hours/week: categorical <3.0, 3.0-8.9, 9.0-17.9, 18.0-26.9, ≥27.0), oral contraceptive use (never, former, current, in NHS II only), postmenopausal hormone use (premenopausal, never, former, current, in NHS and NHS II), alcohol intake (g/d in quintiles), and multivitamin use (yes, no). All covariates (except race, family history of myocardial infarction, baseline hypercholesterolemia and hypertension) were updated every 2 years.

Model 3: model 2 + updated cumulative average of daily intake of total calories (kcals/d in quintiles), full-fat milk (servings/d in categories), bacon (servings/d in categories), red meat (servings/d in categories), other processed meats (servings/d in categories), refined grains (servings/d in categories), fruits (servings/d in categories), vegetables (servings/d in categories), potatoes (servings/d in categories), coffee (servings/d in categories), fruit juices (servings/d in categories), and sugar-sweetened beverages (servings/d in categories).

†*P* values for trend based on continuous egg variable.

Supplemental Table 5: Multivariable adjusted hazard ratios (95% confidence intervals) for incident stroke according to categories of whole egg consumption.

	**Frequency of Egg Consumption***						***P* value for trend**†	**HR (95% CI) per 1 egg per day increase**
	**<1/month**	**1 to <4/month**	**1 to <3/week**	**3 to <5/week**	**5 to <7/week**	**≥1/day**		
				**Nurses' Health Study**				
Cases/Person years	130/83 817	747/405 360	1 862/1 094 983	684/647 309	95/103 795	69/74 065		
Model 1	1.00 (Ref.)	1.04 (0.87, 1.26)	1.00 (0.83, 1.19)	0.98 (0.81, 1.19)	0.96 (0.74, 1.26)	1.13 (0.84, 1.52)	0.73	1.04 (0.90, 1.19)
Model 2	1.00 (Ref.)	1.09 (0.91, 1.32)	1.05 (0.88, 1.26)	1.00 (0.83, 1.21)	0.94 (0.72, 1.22)	1.07 (0.79, 1.44)	0.22	0.98 (0.85, 1.13)
Model 3	1.00 (Ref.)	1.09 (0.90, 1.31)	1.03 (0.86, 1.24)	0.97 (0.79, 1.17)	0.91 (0.69, 1.20)	1.04 (0.77, 1.40)	0.13	0.96 (0.83, 1.12)
				**Nurses' Health Study II**				
Cases/Person years	59/259 216	194/783 545	239/771 290	69/285 454	11/25 679	4/13 287		
Model 1	1.00 (Ref.)	0.96 (0.71, 1.28)	1.12 (0.84, 1.50)	1.13 (0.79, 1.60)	1.65 (0.87, 3.16)	1.15 (0.41, 3.16)	0.07	1.34 (0.90, 2.00)
Model 2	1.00 (Ref.)	0.95 (0.71, 1.27)	1.09 (0.81, 1.45)	1.07 (0.75, 1.53)	1.46 (0.76, 2.80)	1.00 (0.36, 2.76)	0.21	1.18 (0.78, 1.79)
Model 3	1.00 (Ref.)	0.91 (0.67, 1.22)	1.03 (0.76, 1.40)	1.03 (0.71, 1.50)	1.33 (0.68, 2.59)	0.86 (0.31, 2.42)	0.40	1.08 (0.69, 1.68)
				**Health Professionals' Follow-up Study**				
Cases/Person years	182/114 705	404/248 433	641/332 984	368/227 520	83/37 637	62/36 501		
Model 1	1.00 (Ref.)	0.98 (0.82, 1.17)	1.05 (0.88, 1.24)	1.01 (0.84, 1.21)	1.39 (1.07, 1.81)	1.08 (0.81, 1.45)	0.09	1.15 (1.00, 1.31)
Model 2	1.00 (Ref.)	0.96 (0.80, 1.15)	1.01 (0.85, 1.20)	0.95 (0.79, 1.14)	1.31 (1.00, 1.72)	1.01 (0.75, 1.36)	0.33	1.09 (0.95, 1.25)
Model 3	1.00 (Ref.)	0.95 (0.79, 1.15)	0.98 (0.82, 1.18)	0.90 (0.74, 1.11)	1.24 (0.93, 1.66)	0.97 (0.71, 1.33)	0.64	1.07 (0.92, 1.24)
				**Pooled Results**				
Cases/Person years	371/457 738	1 345/1 437 338	2 742/2 199 257	1 121/1 160 283	189/167 111	135/123 853		
Model 1	1.00 (Ref.)	1.02 (0.90, 1.14)	1.03 (0.92, 1.15)	1.01 (0.89, 1.14)	1.16 (0.97, 1.38)	1.12 (0.92, 1.37)	0.16	1.10 (1.00, 1.21)
Model 2	1.00 (Ref.)	1.03 (0.91, 1.15)	1.04 (0.93, 1.16)	0.98 (0.87, 1.11)	1.10 (0.92, 1.31)	1.04 (0.85, 1.28)	0.88	1.04 (0.95, 1.15)
Model 3	1.00 (Ref.)	1.01 (0.90, 1.14)	1.00 (0.89, 1.13)	0.94 (0.82, 1.06)	1.04 (0.86, 1.25)	0.99 (0.81, 1.22)	0.53	1.01 (0.91, 1.12)

*Multivariable adjusted hazard ratios (HR) were estimated from Cox proportional hazards models.

Model 1: adjusted for age (months), and stratified by calendar time (in 2-year intervals) and cohort.

Model 2: model 1 + race (Caucasian, other), family history of myocardial infarction (yes, no), baseline hypercholesterolemia (yes, no), baseline hypertension (yes, no), smoking status (never, former, current), body mass index (kg/m^2: categorical <21.0, 21.0-22.9, 23.0-24.9, 25.0-26.9, 27.0-29.9, 30.0-34.9, ≥35.0), physical activity (metabolic equivalent of task-hours/week: categorical <3.0, 3.0-8.9, 9.0-17.9, 18.0-26.9, ≥27.0), oral contraceptive use (never, former, current, in NHS II only), postmenopausal hormone use (premenopausal, never, former, current, in NHS and NHS II), alcohol intake (g/d in quintiles), and multivitamin use (yes, no). All covariates (except race, family history of myocardial infarction, baseline hypercholesterolemia and hypertension) were updated every 2 years.

Model 3: model 2 + updated cumulative average of daily intake of total calories (kcals/d in quintiles), full-fat milk (servings/d in categories), bacon (servings/d in categories), red meat (servings/d in categories), other processed meats (servings/d in categories), refined grains (servings/d in categories), fruits (servings/d in categories), vegetables (servings/d in categories), potatoes (servings/d in categories), coffee (servings/d in categories), fruit juices (servings/d in categories), and sugar-sweetened beverages (servings/d in categories).

†*P* values for trend based on continuous egg variable.

Supplemental Table 6: Multivariable adjusted hazard ratios (95% confidence intervals) for incident cardiovascular disease according to categories of whole egg consumption: pooled results from the Nurses' Health Study, the Nurses' Health Study II, and the Health Professional's Follow-up Study using fixed-effects meta-analysis.

	**Frequency of Egg Consumption***						***P* value for trend†**	**HR (95% CI) per 1 egg per day increase**
	**<1/month**	**1 to <4/month**	**1 to <3/week**	**3 to <5/week**	**5 to <7/week**	**≥1/day**		
				**Total cardiovascular disease**				
Cases/Person years	1 058/457 330	3 364/1 436 094	6 416/2 197 074	3 042/1 159 160	533/166 930	393/123 726		
Model 3	1.00 (Ref.)	0.98 (0.91, 1.05)	0.98 (0.92, 1.06)	0.93 (0.86, 1.01)	1.04 (0.93, 1.17)	0.94 (0.83, 1.07)	0.23	0.98 (0.92, 1.04)
				**Coronary heart disease**				
Cases/Person years	694/457 612	2 040/1 437 096	3 727/2 199 070	1 937/1 159 993	349/167 056	263/123 830		
Model 3	1.00 (Ref.)	0.96 (0.87, 1.05)	0.96 (0.88, 1.05)	0.92 (0.83, 1.01)	1.02 (0.89, 1.18)	0.93 (0.79, 1.08)	0.35	0.97 (0.90, 1.05)
				**Stroke**				
Cases/Person years	371/457 738	1 345/1 437 338	2 742/2 199 257	1 121/1 160 283	189/167 111	135/123 853		
Model 3	1.00 (Ref.)	1.00 (0.88, 1.13)	1.01 (0.90, 1.14)	0.95 (0.83, 1.08)	1.08 (0.89, 1.30)	1.00 (0.81, 1.24)	0.62	1.02 (0.92, 1.13)

*Multivariable adjusted hazard ratios were estimated from Cox proportional hazards models.

Model 3: stratified by calendar time (in 2-year intervals) and cohort, and adjusted for age (months), race (Caucasian, other), family history of myocardial infarction (yes, no), baseline hypercholesterolemia (yes, no), baseline hypertension (yes, no), smoking status (never, former, current), body mass index (kg/m^2: categorical <21.0, 21.0-22.9, 23.0-24.9, 25.0-26.9, 27.0-29.9, 30.0-34.9, ≥35.0), physical activity (metabolic equivalent of task-hours/week: categorical <3.0, 3.0-8.9, 9.0-17.9, 18.0-26.9, ≥27.0), oral contraceptive use (never, former, current, in NHS II only), postmenopausal hormone use (premenopausal, never, former, current, in NHS and NHS II), alcohol intake (g/d in quintiles), and multivitamin use (yes, no). Model also included updated cumulative average of daily intake of total calories (kcals/d in quintiles), full-fat milk (servings/d in categories), bacon (servings/d in categories), red meat (servings/d in categories), other processed meats (servings/d in categories), refined grains (servings/d in categories), fruits (servings/d in categories), vegetables (servings/d in categories), potatoes (servings/d in categories), coffee (servings/d in categories), fruit juices (servings/d in categories), and sugar-sweetened beverages (servings/d in categories). All covariates (except race, family history of myocardial infarction, baseline hypercholesterolemia and hypertension) were updated every 2 years.

†*P* values for trend based on continuous egg variable.

Supplemental Table 7: Hazard ratios (95% confidence intervals) of incident cardiovascular disease according to seven categories of egg consumption.

	Frequency of Egg Consumption*							***P* value for trend**†
	**<1/month**	**1 to <4/month**	**1 to <3/week**	**3 to <5/week**	**5 to <7/week**	**1 to <2/day**	**≥2/day**	
Cases/Person-years	1 058/457 330	3 364/1 436 094	6 416/2 197 074	3 042/1 159 160	533/166 930	311/101 530	82/22 198	
NHS	1.00 (Ref.)	1.01 (0.89, 1.14)	0.96 (0.84, 1.08)	0.91 (0.79, 1.03)	0.90 (0.75, 1.07)	0.96 (0.78, 1.18)	0.80 (0.51, 1.27)	0.04
NHS II	1.00 (Ref.)	0.84 (0.69, 1.03)	0.95 (0.77, 1.17)	0.76 (0.59, 0.99)	1.32 (0.86, 2.02)	0.58 (0.25, 1.32)	0.89 (0.12, 6.48)	0.80
HPFS	1.00 (Ref.)	0.99 (0.90, 1.09)	1.01 (0.92, 1.11)	0.98 (0.88, 1.09)	1.13 (0.97, 1.32)	0.96 (0.80, 1.15)	1.01 (0.77, 1.33)	0.77
Pooled Results	1.00 (Ref.)	0.98 (0.91, 1.06)	0.98 (0.91, 1.05)	0.92 (0.85, 1.00)	1.01 (0.90, 1.13)	0.93 (0.81, 1.06)	0.91 (0.72, 1.15)	0.20

* All results are stratified by calendar time (in 2-year intervals) and cohort, and adjusted for age (months), race (Caucasian, other), family history of myocardial infarction (yes, no), baseline hypercholesterolemia (yes, no), baseline hypertension (yes, no), smoking status (never, former, current), body mass index (kg/m^2: categorical <21.0, 21.0-22.9, 23.0-24.9, 25.0-26.9, 27.0-29.9, 30.0-34.9, ≥35.0), physical activity (metabolic equivalent of task-hours/week: categorical <3.0, 3.0-8.9, 9.0-17.9, 18.0-26.9, ≥27.0), oral contraceptive use (never, former, current, in NHS II only), postmenopausal hormone use (premenopausal, never, former, current, in NHS and NHS II), alcohol intake (g/d in quintiles), and multivitamin use (yes, no). Model also included updated cumulative average of daily intake of total calories (kcals/d in quintiles), full-fat milk (servings/d in categories), bacon (servings/d in categories), red meat (servings/d in categories), other processed meats (servings/d in categories), refined grains (servings/d in categories), fruits (servings/d in categories), vegetables (servings/d in categories), potatoes (servings/d in categories), coffee (servings/d in categories), fruit juices (servings/d in categories), and sugar-sweetened beverages (servings/d in categories). All covariates (except race, family history of myocardial infarction, baseline hypercholesterolemia and hypertension) were updated every 2 years.

† *P* values for trend are based on continuous egg variable derived from the median egg intake in each category of consumption.

Supplemental Table 8: Multivariable adjusted hazard ratios (95% confidence intervals) for incident cardiovascular disease according to categories of whole egg consumption stratified by key variables.

Variable	Cases/ Person years	Frequency of Egg Consumption*						*P*-trend†	HR (95% CI) per 1 egg per day increase	*P*-interaction‡
		<1/month	1 to <4/month	1 to <3/week	3 to <5/week	5 to <7/week	≥1/day			
Age < 60 years	3 520/ 3 675 627	1.00 (Ref.)	0.96 (0.84, 1.10)	0.96 (0.84, 1.10)	0.94 (0.82, 1.09)	0.92 (0.74, 1.13)	0.87 (0.69, 1.10)	0.26	0.96 (0.85, 1.08)	0.76
Age ≥ 60 years	11 286/ 1 864 686	1.00 (Ref.)	1.02 (0.94, 1.11)	1.03 (0.95, 1.12)	0.97 (0.88, 1.06)	1.11 (0.98, 1.26)	0.99 (0.86, 1.14)	0.73	1.01 (0.94, 1.08)	
BMI < 25 kg/m^2	6 854/ 2 914 229	1.00 (Ref.)	1.00 (0.90, 1.10)	1.00 (0.90, 1.10)	0.94 (0.84, 1.05)	0.89 (0.75, 1.06)	0.93 (0.77, 1.11)	0.07	0.99 (0.90, 1.08)	0.10
BMI ≥ 25 kg/m^2	7 952/ 2 626 083	1.00 (Ref.)	0.97 (0.87, 1.07)	0.95 (0.86, 1.05)	0.91 (0.82, 1.02)	1.09 (0.93, 1.26)	0.93 (0.79, 1.11)	0.87	0.98 (0.90, 1.06)	
< 15 MET-h/week	9 022/ 3 114 238	1.00 (Ref.)	0.96 (0.88, 1.06)	0.96 (0.87, 1.06)	0.88 (0.79, 0.97)	0.93 (0.80, 1.07)	0.90 (0.77, 1.05)	0.03	0.94 (0.87, 1.02)	0.25
≥ 15 MET-h/week	5 784/ 2 426 076	1.00 (Ref.)	0.99 (0.89, 1.10)	0.99 (0.88, 1.10)	1.01 (0.90, 1.14)	1.16 (0.97, 1.39)	0.98 (0.80, 1.20)	0.30	1.06 (0.95, 1.17)	
Never smoker	6 109/ 3 021 417	1.00 (Ref.)	1.05 (0.94, 1.18)	1.07 (0.96, 1.20)	1.03 (0.92, 1.17)	1.15 (0.96, 1.37)	0.99 (0.81, 1.21)	0.86	1.02 (0.92, 1.13)	0.46
Ever smoker	8 697/ 2 518 896	1.00 (Ref.)	0.94 (0.85, 1.03)	0.91 (0.83, 1.00)	0.86 (0.78, 0.95)	0.96 (0.83, 1.11)	0.90 (0.77, 1.05)	0.14	0.97 (0.89, 1.05)	
No hypertension	5 037/ 3 733 397	1.00 (Ref.)	0.97 (0.86, 1.09)	0.93 (0.83, 1.05)	0.83 (0.74, 0.95)	0.88 (0.74, 1.06)	0.76 (0.62, 0.92)	0.005	0.87 (0.78, 0.96)	0.06
Hypertension	9 769/ 1 806 917	1.00 (Ref.)	0.99 (0.90, 1.09)	1.00 (0.91, 1.09)	0.99 (0.89, 1.09)	1.11 (0.96, 1.28)	1.08 (0.92, 1.27)	0.15	1.07 (0.99, 1.16)	
Normal blood cholesterol	6 578/ 3 603 283	1.00 (Ref.)	0.97 (0.87, 1.09)	1.02 (0.92, 1.14)	0.97 (0.86, 1.09)	1.03 (0.88, 1.20)	0.98 (0.83, 1.16)	0.95	1.01 (0.94, 1.10)	0.33
Hypercholesterolemia	8 228/ 1 937 030	1.00 (Ref.)	0.99 (0.90, 1.09)	0.95 (0.86, 1.04)	0.91 (0.81, 1.01)	1.05 (0.89, 1.24)	0.91 (0.74, 1.10)	0.19	0.95 (0.86, 1.05)	
No family history of MI	10 164/ 4 062 467	1.00 (Ref.)	1.00 (0.91, 1.09)	0.98 (0.90, 1.07)	0.93 (0.84, 1.02)	0.99 (0.86, 1.13)	0.94 (0.81, 1.09)	0.14	0.97 (0.90, 1.05)	0.90
Family history of MI	4 642/ 1 477 845	1.00 (Ref.)	0.94 (0.83, 1.06)	0.94 (0.84, 1.06)	0.89 (0.78, 1.02)	1.03 (0.84, 1.25)	0.90 (0.72, 1.13)	0.58	0.98 (0.87, 1.09)	
No statin use	11 039/ 4 839 377	1.00 (Ref.)	1.02 (0.94, 1.11)	1.01 (0.93, 1.10)	0.96 (0.88, 1.05)	1.04 (0.92, 1.18)	0.98 (0.86, 1.13)	0.45	1.00 (0.93, 1.07)	0.54

Statin use	3 767/ 700 937	1.00 (Ref.)	0.88 (0.76, 1.01)	0.89 (0.77, 1.02)	0.85 (0.71, 1.00)	1.07 (0.81, 1.41)	0.73 (0.50, 1.07)	0.53	0.91 (0.76, 1.09)	
AHEI < median	6 796/ 2 782 686	1.00 (Ref.)	1.03 (0.91, 1.17)	1.00 (0.89, 1.13)	0.94 (0.83, 1.07)	1.01 (0.86, 1.18)	0.99 (0.84, 1.18)	0.36	0.99 (0.92, 1.07)	0.41
AHEI > median	8 015/ 2 757 627	1.00 (Ref.)	0.94 (0.86, 1.03)	0.96 (0.87, 1.04)	0.92 (0.83, 1.02)	1.05 (0.88, 1.24)	0.84 (0.68, 1.04)	0.49	0.96 (0.87, 1.06)	
No T2D diagnosis	12 913/ 5 335 087	1.00 (Ref.)	0.99 (0.92, 1.07)	0.98 (0.91, 1.05)	0.91 (0.84, 0.99)	1.01 (0.89, 1.13)	0.93 (0.81, 1.06)	0.08	0.96 (0.90, 1.03)	<0.0001
T2D diagnosis	2 989/ 291 254	1.00 (Ref.)	0.84 (0.69, 1.03)	0.90 (0.74, 1.09)	0.93 (0.76, 1.14)	1.09 (0.85, 1.41)	1.06 (0.81, 1.39)	0.02	1.12 (0.98, 1.28)	

*All results are stratified by calendar time (in 2-year intervals) and cohort, and adjusted for age (months), race (Caucasian, other), family history of myocardial infarction (yes, no), baseline hypercholesterolemia (yes, no), baseline hypertension (yes, no), smoking status (never, former, current), body mass index (kg/m^2: categorical <21.0, 21.0-22.9, 23.0-24.9, 25.0-26.9, 27.0-29.9, 30.0-34.9, ≥35.0), physical activity (metabolic equivalent of task-hours/week: categorical <3.0, 3.0-8.9, 9.0-17.9, 18.0-26.9, ≥27.0), oral contraceptive use (never, former, current, in NHS II only), postmenopausal hormone use (premenopausal, never, former, current, in NHS and NHS II), alcohol intake (g/d in quintiles), and multivitamin use (yes, no). Model also included updated cumulative average of daily intake of total calories (kcals/d in quintiles), full-fat milk (servings/d in categories), bacon (servings/d in categories), red meat (servings/d in categories), other processed meats (servings/d in categories), refined grains (servings/d in categories), fruits (servings/d in categories), vegetables (servings/d in categories), potatoes (servings/d in categories), coffee (servings/d in categories), fruit juices (servings/d in categories), and sugar-sweetened beverages (servings/d in categories). All covariates (except race, family history of myocardial infarction, baseline hypercholesterolemia and hypertension) were updated every 2 years.

† *P* values for trend are based on continuous egg variable derived from the median egg intake in each category of consumption.

‡ *P* values for interaction are calculated using the likelihood ratio test.

AHEI: Alternate Healthy Eating Index; BMI: body mass index; CI: confidence interval; HR: hazard ratio; MET: metabolic equivalent of task; MI: myocardial infarction; T2D: type 2 diabetes.

Supplemental Table 9: Hazard ratios (95% confidence intervals) of incident cardiovascular disease according to categories of total egg consumption, including eggs in mixed foods.

**Cohort**	**Frequency of Egg Consumption***							***P* value for trend†**	**HR (95% CI) per 1 egg per day increase**
	**<1/month**	**1 to <4/month**	**1 to <3/week**	**3 to <5/week**	**5 to <7/week**	**1 to <2/day**	**≥2/day**		
Cases/Person-years	347/150 593	2 559/1 144 369	6 982/2 571 039	3 627/1 312 426	708/208 813	462/125 307	121/27 766		
NHS	1.00 (Ref.)	0.88 (0.73, 1.07)	0.85 (0.70, 1.02)	0.80 (0.66, 0.97)	0.78 (0.62, 0.97)	0.85 (0.66, 1.08)	0.86 (0.55, 1.34)	0.08	0.95 (0.85, 1.05)
NHSII	1.00 (Ref.)	0.70 (0.50, 1.00)	0.76 (0.54, 1.08)	0.74 (0.50, 1.07)	0.81 (0.49, 1.35)	0.74 (0.38, 1.46)	1.31 (0.31, 5.56)	0.67	0.95 (0.70, 1.29)
HPFS	1.00 (Ref.)	1.10 (0.94, 1.29)	1.09 (0.93, 1.27)	1.12 (0.96, 1.32)	1.20 (1.00, 1.44)	1.06 (0.87, 1.28)	1.02 (0.79, 1.32)	0.92	1.00 (0.94, 1.07)
Pooled Results	1.00 (Ref.)	0.98 (0.87, 1.10)	0.97 (0.86, 1.08)	0.95 (0.84, 1.07)	0.98 (0.86, 1.12)	0.94 (0.81, 1.08)	0.90 (0.73, 1.12)	0.27	0.98 (0.93, 1.03)

* All results are stratified by calendar time (in 2-year intervals) and cohort, and adjusted for age (months), race (Caucasian, other), family history of myocardial infarction (yes, no), baseline hypercholesterolemia (yes, no), baseline hypertension (yes, no), smoking status (never, former, current), body mass index (kg/m^2: categorical <21.0, 21.0-22.9, 23.0-24.9, 25.0-26.9, 27.0-29.9, 30.0-34.9, ≥35.0), physical activity (metabolic equivalent of task-hours/week: categorical <3.0, 3.0-8.9, 9.0-17.9, 18.0-26.9, ≥27.0), oral contraceptive use (never, former, current, in NHS II only), postmenopausal hormone use (premenopausal, never, former, current, in NHS and NHS II), alcohol intake (g/d in quintiles), and multivitamin use (yes, no). Model also included updated cumulative average of daily intake of total calories (kcals/d in quintiles), full-fat milk (servings/d in categories), bacon (servings/d in categories), red meat (servings/d in categories), other processed meats (servings/d in categories), refined grains (servings/d in categories), fruits (servings/d in categories), vegetables (servings/d in categories), potatoes (servings/d in categories), coffee (servings/d in categories), fruit juices (servings/d in categories), and sugar-sweetened beverages (servings/d in categories). All covariates (except race, family history of myocardial infarction, baseline hypercholesterolemia and hypertension) were updated every 2 years.

† *P* values for trend are based on continuous egg variable derived from the median egg intake in each category of consumption.

HPFS: Health Professionals' Follow-Up Study; NHS: Nurses' Health Study.

Supplemental Table 10: Characteristics of studies included in the meta-analysis.

Author, year	Study Population	Country	Follow-up (y)	Sample size	Age Range	Assessment of Diet	Ascertainment of Cases	Endpoints (No of cases)	Categories of Exposure	Relative Risks (95% CI)*	Covariates in Multivariable Model
Abdollahi et al. 2019	Kuopio Ischaemic Heart Disease Risk Factor Study	Finland	21.2	1 950 men	42-60 y	Guided 4-day food records, baseline only	Computer linkage to the national hospital discharge and death certificate registers	Stroke (217)	<15 g/d<br>15–26 g/d<br>27–45 g/d<br>>45 g/d	1.00 (reference)<br>1.01 (0.69,1.47)<br>1.10 (0.76,1.61)<br>0.81 (0.54,1.23)	Age, examination year, energy intake, smoking, BMI, leisure-time physical activity, use of hypertension medication, intakes of alcohol, fruits, berries, and vegetables
Díez-Espino et al. 2017	PREDIMED study	Spain	5.8	7 216 men and women	55-80 y	Interviewer-administered FFQ, every year (cumulative average), but HR calculated from baseline intake	Repeated contacts with participants, general practitioners who were responsible for the clinical care of the participants, yearly review of medical records, and consultation of the National Death Index	CVD† (342)	<2/wk<br>2-4/wk<br>>4/wk	1.00 (reference)<br>0.95 (0.75, 1.19)<br>1.22 (0.72, 2.07)	Age, sex, BMI and intervention group, recruitment center, smoking status, physical activity during leisure time, educational status, diabetes, hypertension, hypercholesterolemia, family history of CVD, Mediterranean food pattern, alcohol intake, and total energy intake
Djousse et al. 2008	PHS (Physicians' Health Study)	United States	20	21 327 men	40-86 y	SFFQ, baseline, 24, 48, 72, 96, and 120 months after randomization	Questionnaire were used to gather the information of the occurrence of new medical diagnoses including MI, stroke and confirmed by physicians or medical records, death certificates were obtained for confirmation and review of cause of death	CHD (1 550)	<1/wk<br>1/wk<br>2-4/wk<br>5-6/wk<br>≥7/wk	1.00 (reference)<br>1.12 (0.96, 1.31)<br>1.16 (1.00, 1.36)<br>1.18 (0.93, 1.49)<br>0.90 (0.72, 1.14)	Age, BMI, smoking, history of hypertension, vitamin intake, alcohol, physical activity, breakfast cereal, vegetables, treatment arm, atrial fibrillation, diabetes, hypercholesterolemia, and premature myocardial infarction
								Stroke (1 342)	<1/wk<br>1/wk<br>2-4/wk<br>5-6/wk<br>≥7/wk	1.00 (reference)<br>0.96 (0.82, 1.13)<br>1.06 (0.91, 1.24)<br>1.13 (0.89, 1.42)<br>0.99 (0.80, 1.23)	
Farvid et al. 2017	Golestan Cohort Study	Iran	11	42 403 men and women	36-85 y	Interviewer-administered FFQ baseline only	Reported by family members, friends, or local health workers during annual telephone calls, and a physician visited the house to complete a validated verbal autopsy questionnaire by interviewing the next of kin	CVD death (1 467)	0.00/d<br>0.06/d<br>0.18/d<br>0.48/d<br>Per 3/wk	1.00 (reference)<br>1.01 (0.87, 1.16)<br>0.93 (0.80, 1.08)<br>0.92 (0.79, 1.07)<br>0.92 (0.81, 1.05)	Gender, age, ethnicity, education, marital status, residency, smoking, opium use, alcohol, BMI, systolic blood pressure, occupational physical activity, family history of cancer, wealth score, medication, and energy intake
								CHD death (764)	0.00/d<br>0.06/d<br>0.18/d<br>0.48/d<br>Per 3/wk	1.00 (reference)<br>1.03 (0.84, 1.26)<br>0.99 (0.80, 1.21)<br>0.92 (0.74, 1.14)<br>0.91 (0.77, 1.09)	
								Stroke death (507)	0.00/d<br>0.06/d<br>0.18/d<br>0.48/d<br>Per 3/wk	1.00 (reference)<br>1.00 (0.79, 1.26)<br>0.80 (0.62, 1.04)<br>0.94 (0.73, 1.21)<br>0.94 (0.75, 1.17)	
Goldberg et al. 2014	Northern Manhattan Study	United States	11	2 669 men and women	>40 y	Interviewer-administered	Detected through ongoing hospital surveillance of	CVD (719)	<1/mo<br>1/mo<br>2-3/mo	1.00 (reference)<br>0.94 (0.75, 1.16)<br>0.85 (0.66, 1.09)	Age, sex, race/ethnicity, BMI, diabetes, hypertension, LDL-C,

						FFQ, baseline only	admission and discharge data from all area hospitals		1/wk	0.96 (0.79, 1.18)	HDL-C, TG, cholesterol lowering medication, moderate alcohol use, moderate-heavy physical activity, smoking, high-school completion, energy intake, Mediterranean diet score, family history of stroke in siblings, family history of MI in siblings, consumption of saturated and unsaturated fats, carbohydrates, and proteins
									≥2/wk	1.03 (0.67, 1.60)	
									Per 1 egg/wk	1.05 (0.95, 1.16)	
								Stroke (266)	<1/mo	1.00 (reference)	
									1/mo	0.97 (0.69, 1.37)	
									2-3/mo	0.76 (0.50, 1.14)	
									1/wk	0.83 (0.60, 1.16)	
									≥2/wk	1.18 (0.60, 2.30)	
									Per 1 egg/wk	1.04 (0.88, 1.22)	
								CHD (226)	<1/mo	1.00 (reference)	
									1/mo	0.83 (0.57, 1.22)	
									2-3/mo	0.66 (0.40, 1.06)	
									1/wk	1.09 (0.77, 1.55)	
									≥2/wk	0.81 (0.34, 1.93)	
									Per 1 egg/wk	1.04 (0.87, 1.26)	
Guo et al. 2018	CAPS	United Kingdom	22.8	1 781 men	45-59 y	SFFQ, updated every 5-years	Hospital and general practitioner database, confirmed by two independent expert clinicians and an epidemiologist, including computed tomography, radiological and pathological information and National Registry	CVD (715)	0-1/wk	1.00 (reference)	Age, BMI, total energy intake, alcohol consumption, smoking, energy expenditure, social class, family history of myocardial infarction, diabetes mellitus, sugar intake, fruit consumption, red meat consumption, and fiber intake
									1-2/wk	0.98 (0.76, 1.26)	
									2-3/wk	1.14 (0.89, 1.46)	
									3-5/wk	1.01 (0.77, 1.33)	
									≥5/wk	1.25 (0.94, 1.66)	
								Stroke (248)	0-1/wk	1.00 (reference)	
									1-2/wk	1.01 (0.65, 1.56)	
									2-3/wk	1.00 (0.64, 1.55)	
									3-5/wk	1.15 (0.72, 1.84)	
									≥5/wk	1.60 (1.00, 2.57)	
								CHD (477)	0-1/wk	1.00 (reference)	
									1-2/wk	0.97 (0.72, 1.31)	
									2-3/wk	1.14 (0.85, 1.52)	
									3-5/wk	1.01 (0.72, 1.4)	
									≥5/wk	0.91 (0.64, 1.31)	
Houston et al. 2011	Health, Aging and Body Composition (Health ABC) Study	United States	9	1 600 men and women (without type 2 diabetes at baseline)	70-79 y	Interviewer-administered FFQ, at year 2	Annual in-person clinic exams, semi-annual phone interviews, medical record and death certificate	CVD (158)	<1/wk	1.00 (reference)	Age, sex, race, education, field center, smoking, alcohol use, physical activity, BMI, total energy intake, protein intake, fiber intake, multivitamin use, supplemental vitamin E use, statin use, aspirin use, oral estrogen use (women), prevalent hypertension, and saturated fatty acid intake
									1-2/wk	1.03 (0.71, 1.49)	
									≥3/wk	1.38 (0.88, 2.16)	
Jang et al. 2018	Korean Genome and Epidemiology Study	Korea	7.3	9 248 men and women	40-69 y	SFFQ, baseline and second follow-up	Identified through biennial questionnaires, and all reported cases were confirmed by trained staff during personal interviews	CVD (570)	0.1/wk	1.00 (reference)	Age, sex, educational level, residential area, monthly household income, alcohol drinking, smoking in pack-years, physical activity level, dietary supplement use, history of hypertension and dyslipidemia, and intakes of total energy, total vegetables, total fruits, red meat, fiber, vitamin E, and BMI
									0.7/wk	1.27 (0.99, 1.61)	
									1.6/wk	1.23 (0.95, 1.60)	
									4.2/wk	1.14 (0.87, 1.49)	

Key et al. 2019	EPIC	Denmark, Norway, Sweden, France, Netherlands, UK, Greece, Italy, Spain, Germany	12.6	409 885 men and women	21-83 y	FFQ, baseline only	Record linkage to morbidity or hospital registries, and self-reports followed by confirmation with medical records; vital status collected from mortality registries at the regional or national level or by active follow-up of study participants and next of kin	CHD (7 198)	4 g/d<br>9 g/d<br>15 g/d<br>22 g/d<br>40 g/d<br>Per 20 g/d	1.00 (reference)<br>0.96 (0.89-1.04)<br>0.97 (0.90-1.05)<br>1.02 (0.94-1.09)<br>0.93 (0.86-1.01)<br>0.93 (0.88, 0.99)	Age, smoking status, number of cigarettes per day, history of diabetes, previous hypertension, prior hyperlipidemia, Cambridge physical activity index, employment status, level of education completed, BMI, current alcohol consumption, and intakes of energy, fruit and vegetables combined, sugars and fibre from cereals, sex, and EPIC centre;<br>Dose response analysis further adjusted for intakes of red and processed meat, poultry meat, white fish, fatty fish, milk, yogurt, and cheese
Larsson et al. 2015	Cohort of Swedish Men	Sweden	13	37 766 men	45-79 y	SFFQ, baseline only	Confirmed by Swedish National Patient and Cause of Death Registers	CHD (3 262)	0–3/mo<br>1–2/wk<br>3–6/wk<br>1.2/d	1.00 (reference)<br>0.98 (0.90, 1.05)<br>0.95 (0.84, 1.08)<br>1.03 (0.84, 1.27)	Age, education, family history of CHD before 60 y of age, smoking status and pack-years of smoking, aspirin use, walking/cycling, exercise, BMI, history of hypertension, hypercholesterolemia, and diabetes, and intakes of total energy, alcohol, fruits and vegetables, and processed meat
								Ischemic stroke (2 039)	0–3/mo<br>1–2/wk<br>3–6/wk<br>1.2/d	1.00 (reference)<br>0.91 (0.83, 1.00)<br>1.07 (0.92, 1.24)<br>0.87 (0.66, 1.14)	
								Hemorrhagic stroke (405)	0–3/mo<br>1–2/wk<br>3–6/wk<br>1.2/d	1.00 (reference)<br>0.91 (0.73, 1.14)<br>1.04 (0.74, 1.45)<br>1.05 (0.59, 1.88)	
	Swedish Mammography Cohort	Sweden	13	32 805 women	49-83 y	SFFQ, baseline only	Confirmed by Swedish National Patient and Cause of Death Registers	CHD (1 504)	0–3/mo<br>1–2/wk<br>3–6/wk<br>1.1/d	1.00 (reference)<br>0.94 (0.84, 1.05)<br>1.03 (0.86, 1.24)<br>0.85 (0.59, 1.23)	Age, education, family history of CHD before 60 y of age, smoking status and pack-years of smoking, aspirin use, walking/cycling, exercise, BMI, history of hypertension, hypercholesterolemia, and diabetes, and intakes of total energy, alcohol, fruits and vegetables, and processed meat
								Ischemic stroke (1 561)	0–3/mo<br>1–2/wk<br>3–6/wk<br>1.1/d	1.00 (reference)<br>1.06 (0.95, 1.19)<br>1.07 (0.90, 1.28)<br>1.06 (0.76, 1.47)	
								Hemorrhagic stroke (294)	0–3/mo<br>1–2/wk<br>3–6/wk<br>1.1/d	1.00 (ref)<br>1.06 (0.82, 1.36)<br>0.80 (0.52, 1.25)<br>0.96 (0.44, 2.12)	
Mann et al. 1997	Vegetarian Society	UK	13.3	9 980 men and women	16-79 y	Self-administered SFFQ<br>Baseline only	Confirmed by the National Health Service Central Register	CHD death (64)	<1/wk<br>1–5/wk<br>≥6/wk	1.00 (reference)<br>1.28 (0.59, 2.79)<br>2.68 (1.19, 6.02)	Age, sex, smoking, and social class
Misirli et al. 2012	EPIC-Greece Cohort	Greece	10.6	23 601 men and women	NA	Interviewer-administered SFFQ, baseline only	Self-reported and confirmed by pathology reports, medical records, discharge	Stroke (395)	Per 1 SD increment (11 g/d)	1.07 (0.98, 1.18)	Sex, BMI, age, education, smoking status, physical activity, diabetes, hypertension, energy intake

							diagnoses, or death certificates				
Nakamura et al. 2004	NIPPON DATA80	Japan	14	4 077 men	≥30 y	Self-administered FFQ baseline only	Confirmed by National Registry (computer matching of data from the National Vital Statistics)	CHD death (39)	1/d<br>0.5 d<br>1–2/wk<br>Seldom	1.00 (reference)<br>1.49 (0.63, 3.48)<br>1.71 (0.78, 3.76)<br>1.18 (0.26, 5.42)	Age, serum creatinine, total cholesterol, blood glucose, BMI, systolic and diastolic BP, use of BP-lowering drugs, cigarette smoking, and alcohol intake
								Stroke death (112)	≥2/d<br>1/d<br>0.5 d<br>1–2/wk<br>Seldom	0.25 (0.03, 1.81)<br>1.00 (reference)<br>1.10 (0.68, 1.76)<br>1.09 (0.69, 1.72)<br>0.93 (0.36, 2.40)	
				5 186 women	≥30 y	Self-administered FFQ baseline only	Confirmed by National Registry (computer matching of data from the National Vital Statistics)	CHD death (41)	≥2/d<br>1/d<br>0.5 d<br>1–2/wk<br>Seldom	1.27 (0.16, 9.80)<br>1.00 (reference)<br>0.78 (0.35, 1.82)<br>0.64 (0.28, 1.44)<br>1.42 (0.56, 3.62)	Age, serum creatinine, total cholesterol, blood glucose, BMI, systolic and diastolic BP, use of BP-lowering drugs, cigarette smoking, and alcohol intake
								Stroke death (107)	≥2/d<br>1/d<br>1/2 d<br>1–2/wk<br>Seldom	1.22 (0.29, 5.17)<br>1.00 (reference)<br>1.46 (0.89, 2.40)<br>0.79 (0.47, 1.33)<br>0.78 (0.35, 1.73)	
Nakamura et al. 2006	Japan Public Health Center-based prospective study	Japan	10.2	90 735 men and women	40-69 y	Self-administered questionnaire, baseline only	Confirmed by medical records, letter, telephone or death certificate	CHD (3 587)	<1/wk<br>1-2/wk<br>3-4/wk<br>Almost daily	1.19 (0.86, 1.64)<br>1.00 (0.77, 1.3)<br>1.00 (0.79, 1.26)<br>1.00 (reference)	Age, sex, BMI, hypertension, diabetes, use of cholesterol lowering drugs, smoking, alcohol drinking, whether participants intended to avoid cholesterol rich diets, consumption of meat, fish, vegetables, and fruits, and cohort effects
Nakamura et al. 2018	NIPPON DATA90	Japan	15	4 686 women	≥30 y	Self-administered questionnaire baseline only	Identified through the National Vital Statistics	CVD death (183)	<1/week<br>1-2/week<br>0.5/d<br>1/d<br>≥2/day	1.09 (0.60, 1.97)<br>1.16 (0.81, 1.67)<br>0.92 (0.61, 1.38)<br>1.00 (reference)<br>1.24 (0.38, 4.10)	Age, BMI, BMIxBMI, hypertension, diabetes, cigarette smoking, alcohol drinking, dyslipidemia therapy, intake of fiber, meat, and sodium
Qin et al. 2018	CKB	China	8.9	461 213 men and women	30-79 y	Interviewer-administered FFQ, baseline, second and third survey	Obtained regularly via local disease and death registries, checked against the national health insurance system with electronic linkage to all hospitalizations, or ascertained through active follow-up	CVD (83 977)	0.29/d<br>0.36/d<br>0.46/d<br>0.56/d<br>0.76/d<br>Per 1 egg/wk	1.00 (reference)<br>0.97 (0.95, 1.00)<br>0.92 (0.90, 0.94)<br>0.90 (0.87, 0.93)<br>0.89 (0.87, 0.92)<br>0.97 (0.96, 0.98)	Age at recruitment, sex, education level, household income, marital status, alcohol consumption, tobacco smoking, physical activity in MET-hours/day, BMI, waist to hip ratio, prevalent hypertension, use of aspirin, family history of CVD, multivitamin supplementation, and dietary pattern
								CHD (30 169)	0.29/d<br>0.36/d<br>0.46/d<br>0.56/d<br>0.76/d<br>Per 1 egg/wk	1.00 (reference)<br>0.95 (0.91, 0.99)<br>0.92 (0.88, 0.96)<br>0.86 (0.81, 0.91)<br>0.88 (0.84, 0.93)<br>0.97 (0.95, 0.98)	
								Hemorrhagic stroke (7 078)	0.29/d<br>0.36/d<br>0.46/d<br>0.56/d<br>0.76/d	1.00 (reference)<br>0.86 (0.79, 0.93)<br>0.82 (0.76, 0.88)<br>0.77 (0.70, 0.86)<br>0.74 (0.67, 0.82)	

									Per 1 egg/wk	0.92 (0.90, 0.95)	
								Ischemic stroke (27 745)	0.29/d<br>0.36/d<br>0.46/d<br>0.56/d<br>0.76/d<br>Per 1 egg/wk	1.00 (reference)<br>0.98 (0.94, 1.03)<br>0.95 (0.91, 1.00)<br>0.95 (0.90, 1.00)<br>0.90 (0.85, 0.95)<br>0.97 (0.96, 0.98)	
Qureshi et al. 2007	NHANES-I	United States	20	9 734 men and women	25-74 y	Self-administered nutritional questionnaire, baseline only	Confirmed by medical records or death certificate	Stroke (655)	<1/wk<br>1–6/wk<br>>6/wk	1.00 (reference)<br>0.90 (0.70, 1.00)<br>0.90 (0.70, 1.10)	Age, sex, race/ethnicity, systolic blood pressure, diabetes, serum cholesterol, smoking, BMI, education
								Ischemic stroke (591)	<1/wk<br>1–6/wk<br>>6/wk	1.00 (reference)<br>0.80 (0.70, 1.00)<br>0.90 (0.70, 1.10)	
								CHD (1 584)	<1/wk<br>1–6/wk<br>>6/wk	1.00 (reference)<br>1.00 (0.90, 1.10)<br>1.10 (0.90, 1.30)	
Sauvaget et al. 2003	Life Span Study	Japan	16	37 130 men and women	34-103 y	Self-administered FFQ baseline only	Confirmed by the nationwide family registration system of Japan	Stroke death (1 462)	Never<br>≤1 time/week<br>2–4 time/week<br>Almost daily	1.00 (reference)<br>0.75 (0.55, 1.01)<br>0.77 (0.57, 1.03)<br>0.70 (0.51 0.95)	Sex and birth cohort, and adjusted for city, radiation dose, self-reported body mass index, smoking status, alcohol habits, education level, history of diabetes, or hypertension.
Scrafford et al. 2011	NHANES III	United States	8.8	6 833 men	>17 y	Self-administered FFQ baseline only	Linking death records from National Death Index	CHD death (198)	0.27/wk<br>1.93/wk<br>7.54/wk	1.00 (reference)<br>1.26 (0.79, 2.00)<br>1.13 (0.61, 2.11)	Age, energy, marital status, education status, race-ethnicity, BMI, diabetes, hypertension, and alcohol intake
								Stroke death (63)	0.27/wk<br>1.93/wk<br>7.54/wk	1.00 (reference)<br>1.00 (0.49, 2.02)<br>0.27 (0.10, 0.73)	
			8.9	8 113 women	>17 y	Self-administered FFQ baseline only	Linking death records from National Death Index	CHD death (168)	0.24/wk<br>1.79/wk<br>7.41/wk	1.00 (reference)<br>1.12 (0.66, 1.89)<br>0.92 (0.27, 3.11)	Age, energy, marital status, education status, race-ethnicity, BMI, diabetes, hypertension, and alcohol intake
								Stroke death (74)	0.24/wk<br>1.79/wk<br>7.41/wk	1.00 (reference)<br>0.93 (0.46, 1.90)<br>1.03 (0.25, 4.22)	
van den Brandt et al. 2019	NLCS	Netherlands	10	120 852 men and women	55-69 y	Self-administered FFQ baseline only	Death certificates linkage to statistics Netherlands	CVD Death (2 985)	0 g/day<br>7.1 g/day<br>14.2 g/day<br>21.4 g/day<br>Per 50g/day	1.00 (reference)<br>0.89 (0.69, 1.16)<br>0.90 (0.70, 1.16)<br>0.92 (0.71, 1.19)<br>0.92 (0.70, 1.20)	age at baseline, sex, cigarette smoking status, number of cigarettes smoked per day, and years of smoking, history of physician-diagnosed hypertension and diabetes, body height, BMI, non-occupational physical activity, highest level of education, intake of alcohol, vegetables and fruit , energy, use of nutritional supplements, and, in women, postmenopausal HRT
Virtanen et al. 2016	Kuopio Ischaemic Heart Disease Risk Factor Study	Finland	20.8	1 032 men	42-60 y	Guided 4-day food records, baseline only	Computer linkage to the national hospital discharge and death certificate registers	CHD (230)	11 g/d<br>26 g/d<br>52 g/d	1.00 (reference)<br>0.96 (0.69, 1.34)<br>1.18 (0.85, 1.66)	Age, examination year, energy intake, smoking, BMI, diabetes, hypertension, leisure-time

											physical activity, coronary artery disease history in close relatives, education, and intakes of alcohol, fruits, berries, vegetables, fiber, polyunsaturated fatty acids, and saturated fatty acids
Wang et al. 2016	Linxian NIT	China	26	2 445 men and women	40-69 y	Interviewer-administered FFQ baseline only	Doctor visits/ Hospital records reviews/ National registry	CHD death (355)	Per 4 times/month	1.00 (0.95, 1.06)	Age, sex, commune, smoking, drinking, season and BMI
								Stroke death (452)	Per 4 times/month	1.00 (0.96, 1.06)	
Xu et al. 2018	Guangzhou Biobank Cohort Study	China	9.8	28 024 men and women	≥50 y	FFQ baseline only	Obtained via record linkage with the Guangzhou Center for Disease Control and Prevention (GCDC)	CVD death (873)	<1/week<br>1-2/week<br>3-4/week<br>5-6/week<br>≥7/week	1.00 (reference)<br>0.92 (0.77, 1.10)<br>0.96 (0.80, 1.14)<br>0.81 (0.58, 1.14)<br>0.99 (0.76, 1.27)	Sex, age, education, occupation, family income, smoking status, physical activity, alcohol drinking, self-rated health and chronic disease history (diabetes, hypertension and dyslipidemia)
								CHD death (388)	<1/week<br>1-2/week<br>3-4/week<br>5-6/week<br>≥7/week	1.00 (reference)<br>0.86 (0.66, 1.13)<br>1.03 (0.79, 1.34)<br>0.75 (0.44, 1.27)<br>0.92 (0.63, 1.36)	
								Stroke death (341)	<1/week<br>1-2/week<br>3-4/week<br>5-6/week<br>≥7/week	1.00 (reference)<br>0.99 (0.75, 1.30)<br>0.90 (0.68, 1.20)<br>0.81 (0.47, 1.38)<br>0.88 (0.57, 1.35)	
Yaemsiri et al. 2012	WHI-OS	United States	7.6	87 025 women	50-79 y	Self-administered FFQ, baseline and at follow-up visit 3 years later	Self-report during annual medical history; medical charts, brain imaging, or death certificates	Ischemic stroke (1 049)	Per 1 medium serving/d	0.86 (0.55, 1.33)	Age, race, education, family income, years as a regular smoker, hormone replacement therapy use, total MET-hours per week, alcohol intake, history of CHD, history of atrial fibrillation, history of diabetes, aspirin use, use of antihypertensive medication, use of cholesterol-lowering medication, BMI, systolic blood pressure, intakes of total energy, dietary vitamin E, fruits, vegetables, and fibers
Zazpe et al. 2011	The SUN Project	Spain	5.8	14 185 men and women	20-90 y	Self-administered questionnaire, baseline only	Self-reported questionnaire and confirmed by medical record	CVD (91)	<1/wk<br>1/wk<br>2-4/wk<br>>4/wk	1.00 (reference)<br>0.78 (0.36, 1.70)<br>1.00 (0.51, 1.97)<br>1.10 (0.46, 2.63)	Age, sex, total energy intake, adherence to the Mediterranean food pattern, alcohol intake, BMI, smoking status, physical activity, family history of CVD, diabetes, hypertension, and hypercholesterolemia

Zhong et al. 2019	The Lifetime Risk Pooling Project, 6 cohorts (ARIC, CARDIA, FHS, FOS, JHS, MESA)	United States	17.5	29 615 men and women	NA	FFQs, baseline only	Adjudicated cause of death by review of medical records and/or autopsies by study investigators	CVD (5 400)	0/d	1.00 (reference)	Age, sex, race/ethnicity, education, total energy, smoking status, smoking pack-years, cohort-specific physical activity z- score, alcohol consumption, and use of hormone replacement therapy, BMI, diabetes status, systolic blood pressure, use of anti-hypertensive medications, high density lipoprotein cholesterol, non-HDL-C, and use of lipid-lowering medications
									<0.5/d	1.06 (0.98, 1.14)	
									0.5-1/d	1.05 (0.92, 1.20)	
									1-2/d	1.17 (1.04, 1.31)	
									≥2/d	1.29 (1.04, 1.59)	
								CHD (2 088)	Per 0.5/d	1.07 (1.01, 1.12)	
								Stroke (1 302)	Per 0.5/d	1.10 (1.03, 1.18)	
Drouin-Chartier et al. (current)	NHS	United States	32	83 349 women	30-55 y	Self-administered SFFQ, every 4 years	Self-report with or without validation with medical records	CVD (7 411)	<1/mo	1.00 (reference)	Age, calendar time, smoking status, BMI, physical activity, postmenopausal hormone use, race, family history of myocardial infarction, baseline high blood cholesterol, baseline high blood pressure, alcohol intake, multivitamin use, daily intake of total calories, full-fat milk, bacon, red meat, other processed meats, refined grains, fruits, vegetables, potatoes, coffee, fruit juices, and sugar-sweetened beverages
									1-<4/mo	1.01 (0.89, 1.14)	
									1-<3/wk	0.96 (0.84, 1.08)	
									3-<5/wk	0.91 (0.79, 1.03)	
									5-<7/wk	0.90 (0.75, 1.07)	
									≥1/d	0.93 (0.77, 1.14)	
									Per 1 egg/d	0.94 (0.85, 1.04)	
								CHD (3 896)	<1/mo	1.00 (reference)	
									1-<4/mo	0.94 (0.79, 1.12)	
									1-<3/wk	0.89 (0.76, 1.05)	
									3-<5/wk	0.85 (0.71, 1.01)	
									5-<7/wk	0.88 (0.69, 1.11)	
									≥1/d	0.85 (0.65, 1.11)	
									Per 1 egg/d	0.92 (0.80, 1.05)	
								Stroke (3 587)	<1/mo	1.00 (reference)	
									1-<4/mo	1.09 (0.90, 1.31)	
									1-<3/wk	1.03 (0.86, 1.24)	
									3-<5/wk	0.97 (0.79, 1.17)	
									5-<7/wk	0.91 (0.69, 1.20)	
									≥1/d	1.04 (0.77, 1.40)	
									Per 1 egg/d	0.96 (0.83, 1.12)	
	NHS II	United States	22	90 214 women	25-44 y	Self-administered SFFQ, every 4 years	Self-report with or without validation with medical records	CVD (1 225)	<1/mo	1.00 (reference)	Age, calendar time, smoking status, BMI, physical activity, oral contraceptive use, postmenopausal hormone use, race, family history of myocardial infarction, baseline high blood cholesterol, baseline high blood pressure, alcohol intake, multivitamin use, daily intake of total calories, full-fat milk, bacon, red meat, other processed meats, refined grains, fruits, vegetables, potatoes, coffee, fruit juices, and sugar-sweetened beverages
									1-<4/mo	0.84 (0.69, 1.03)	
									1-<3/wk	0.95 (0.77, 1.17)	
									3-<5/wk	0.76 (0.59, 0.99)	
									5-<7/wk	1.32 (0.86, 2.02)	
									≥1/d	0.61 (0.28, 1.31)	
									Per 1 egg/d	0.97 (0.71, 1.33)	
								CHD (653)	<1/mo	1.00 (reference)	
									1-<4/mo	0.77 (0.59, 1.01)	
									1-<3/wk	0.86 (0.65, 1.14)	
									3-<5/wk	0.56 (0.39, 0.81)	
									5-<7/wk	1.31 (0.75, 2.27)	
									≥1/d	0.43 (0.13, 1.38)	
									Per 1 egg/d	0.91 (0.59, 1.42)	
								Stroke (576)	<1/mo	1.00 (reference)	
									1-<4/mo	0.91 (0.67, 1.22)	
									1-<3/wk	1.03 (0.76, 1.40)	
									3-<5/wk	1.03 (0.71, 1.50)	
									5-<7/wk	1.33 (0.68, 2.59)	

									≥1/d<br>Per 1 egg/d	0.86 (0.31, 2.42)<br>1.08 (0.69, 1.68)	
	HPFS	United States	26	42 055 men	40-75 y	Self-administered SFFQ, every 4 years	Self-report with or without validation with medical records	CVD (6 170)	<1/mo<br>1-<4/mo<br>1-<3/wk<br>3-<5/wk<br>5-<7/wk<br>≥1/d<br>Per 1 egg/d	1.00 (reference)<br>0.99 (0.90, 1.09)<br>1.01 (0.92, 1.11)<br>0.98 (0.88, 1.09)<br>1.13 (0.97, 1.32)<br>0.97 (0.82, 1.14)<br>1.01 (0.93, 1.10)	Age, calendar time, smoking status, BMI, physical activity, family history of myocardial infarction, baseline high blood cholesterol, baseline high blood pressure, alcohol intake, multivitamin use, daily intake of total calories, full-fat milk, bacon, red meat, other processed meats, refined grains, fruits, vegetables, potatoes, coffee, fruit juices, and sugar-sweetened beverages
								CHD (4 461)	<1/mo<br>1-<4/mo<br>1-<3/wk<br>3-<5/wk<br>5-<7/wk<br>≥1/d<br>Per 1 egg/d	1.00 (reference)<br>1.00 (0.89, 1.12)<br>1.02 (0.91, 1.14)<br>1.01 (0.89, 1.14)<br>1.09 (0.91, 1.31)<br>0.99 (0.82, 1.20)<br>1.00 (0.91, 1.10)	
								Stroke (1 740)	<1/mo<br>1-<4/mo<br>1-<3/wk<br>3-<5/wk<br>5-<7/wk<br>≥1/d<br>Per 1 egg/d	1.00 (reference)<br>0.95 (0.79, 1.15)<br>0.98 (0.82, 1.18)<br>0.90 (0.74, 1.11)<br>1.24 (0.93, 1.66)<br>0.97 (0.71, 1.33)<br>1.07 (0.92, 1.24)	
**Studies among individuals with type 2 diabetes**											
Díez-Espino et al. 2017	PREDIMED study	Spain	5.8	3 527 men and women with type 2 diabetes	55-80 y	Interviewer-administered FFQ, every year (cumulative average), but HR calculated from baseline intake	Repeated contacts with participants, general practitioners who were responsible for the clinical care of the participants, yearly review of medical records, and consultation of the National Death Index	CVD (225)	<2/wk<br>2-4/wk<br>>4/wk	1.00 (reference)<br>0.86 (0.65, 1.14)<br>1.33 (0.72, 2.46)	Age, sex, BMI, intervention group, recruitment center, smoking status, physical activity during leisure time, educational status, diabetes, hypertension, hypercholesterolemia, family history of CVD, Mediterranean food pattern, alcohol intake, and total energy intake
Houston et al. 2011‡	Health, Aging and Body Composition (Health ABC) Study	United States	9	341 men and women with type 2 diabetes	70-79 y	Interviewer-administered FFQ, at year 2	Annual in-person clinic exams, semi-annual phone interviews, medical record and death certificate	CVD (45)	<1/wk<br>1-2/wk<br>≥3/wk	1.00 (reference)<br>3.33 (1.18, 9.41)<br>5.02 (1.63, 15.52)	Age, sex, race, education, field center, smoking, alcohol use, physical activity, BMI, total energy intake, protein intake, fiber intake, multivitamin use, supplemental vitamin E use, statin use, aspirin use, oral estrogen use (women), prevalent hypertension, and saturated fatty acids
Jang et al. 2018	Korean Genome and Epidemiology Study	Korea	7.3	615 men and women with type 2 diabetes	40-69 y	SFFQ, baseline and second follow-up	Identified through biennial questionnaires, and all reported cases were confirmed by trained staff during personal interviews	CVD (79)	0.1/wk<br>0.7/wk<br>1.6/wk<br>4.2/wk	1.00 (reference)<br>1.72 (0.81, 3.64)<br>3.70 (1.65, 8.30)<br>2.81 (1.25, 6.30)	Age, sex, educational level, residential area, monthly household income, alcohol drinking, smoking in pack-years, physical activity level, dietary supplement use, history of hypertension and

											dyslipidemia, and intakes of total energy, total vegetables, total fruits, red meat, fiber, vitamin E, and BMI
Larsson et al. 2015	Cohort of Swedish Men and Swedish Mammography Cohort	Sweden	13	Number of men and women with type 2 diabetes not available.	45-79 y	SFFQ, baseline only	Confirmed by Swedish National Patient and Cause of Death Registers	CHD (612)	0–3/mo<br>1–2/wk<br>3–6/wk<br>≥1.15/d	1.00 (reference)<br>0.98 (0.80, 1.21)<br>0.92 (0.66, 1.28)<br>0.95 (0.57, 1.60)	Age, education, family history of MI before 60 y of age, smoking status and pack-years of smoking, aspirin use, walking/cycling, exercise, BMI, history of hypertension, hypercholesterolemia, and diabetes, and intakes of total energy, alcohol, fruits and vegetables, and processed meat
								Ischemic stroke (455)	0–3/mo<br>1–2/wk<br>3–6/wk<br>≥1.15/d	1.00 (reference)<br>1.03 (0.80, 1.33)<br>0.88 (0.60, 1.29)<br>0.83 (0.46, 1.48)	
								Hemorrhagic stroke (44)	0–3/mo<br>1–2/wk<br>3–6/wk<br>≥1.15/d	1.00 (reference)<br>0.38 (0.11, 1.25)<br>1.63 (0.39, 6.74)<br>-	
Qureshi et al. 2007	NHANES-I	United States	20	349 men and women with type 2 diabetes	25-74 y	Self-administered nutritional questionnaire, baseline only	Confirmed by medical records or death certificate	Stroke (57)	<1/wk<br>1–6/wk<br>≥7/wk	1.00 (reference)<br>1.10 (0.50, 2.50)<br>0.60 (0.20, 1.50)	Age, sex, race/ethnicity, systolic blood pressure, diabetes, serum cholesterol, smoking, BMI, education
								Ischemic stroke (51)	<1/wk<br>1–6/wk<br>≥7/wk	1.00 (reference)<br>1.10 (0.40, 2.10)<br>0.50 (0.20, 1.40)	
								CHD (132)	<1/wk<br>1–6/wk<br>≥7/wk	1.00 (reference)<br>1.20 (0.70, 2.30)<br>1.90 (1.00, 3.50)	
Scrafford et al 2011	NHANES III	United States	8.8	743 men and women	>17 y	Self-administered questionnaire, FFQ baseline only	Linking death records from National Death Index	CHD death (66)	0.22/wk<br>1.87/wk<br>7.21/wk	1.00 (reference)<br>0.63 (0.24, 1.64)<br>0.97 (0.40, 2.39)	Age, sex, and energy intake
								Stroke death (24)	0.22/wk<br>1.87/wk<br>7.21/wk	1.00 (reference)<br>1.79 (0.93, 6.30)<br>0.32 (0.07, 1.42)	
Trichopoulou et al 2006	EPIC-Greece	Greece	4.5	1 013 men and women		Interviewer-administered FFQ baseline only	Based on death certificate information.	CVD Death (46)	Per 10g/d	1.54 (1.20, 1.97)	Gender, age, educational level, smoking, waist-to-height, hip circumference, MET score, treatment with insulin, treatment for hypertension at enrolment, and treatment for hypercholesterolaemia at enrolment and other indicated food groups.
Drouin-Chartier et al. (current)	NHS	United States	32	10 936 women	30-55 y	Self-administered SFFQ, every 4 years	Self-report with or without validation with medical records	CVD (1 725)	<1/mo<br>1-<4/mo<br>1-<3/wk<br>3-<5/wk<br>5-<7/wk<br>≥1/d<br>Per 1 egg/d	1.00 (reference)<br>0.84 (0.61, 1.16)<br>0.99 (0.73, 1.34)<br>1.06 (0.77, 1.45)<br>1.23 (0.85, 1.78)<br>1.26 (0.84, 1.88)<br>1.29 (1.08, 1.54)	Age, calendar time, smoking status, BMI, physical activity, postmenopausal hormone use, race, family history of myocardial infarction, baseline high blood cholesterol, baseline high blood pressure, alcohol intake, multivitamin use, daily intake of total calories, full-fat milk,

										bacon, red meat, other processed meats, refined grains, fruits, vegetables, potatoes, coffee, fruit juices, and sugar-sweetened beverages
NHS II	United States	22	6 414 women	25-44 y	Self-administered SFFQ, every 4 years	Self-report with or without validation with medical records	CVD (226)	<1/mo<br>1-<4/mo<br>1-<3/wk<br>3-<5/wk<br>5-<7/wk<br>≥1/d<br>Per 1 egg/d	1.00 (reference)<br>0.44 (0.26, 0.75)<br>0.44 (0.26, 0.74)<br>0.39 (0.21, 0.73)<br>0.66 (0.25, 1.73)<br>0.57 (0.17, 1.91)<br>1.02 (0.52, 2.00)	Age, calendar time, smoking status, BMI, physical activity, oral contraceptive use, postmenopausal hormone use, race, family history of myocardial infarction, baseline high blood cholesterol, baseline high blood pressure, alcohol intake, multivitamin use, daily intake of total calories, full-fat milk, bacon, red meat, other processed meats, refined grains, fruits, vegetables, potatoes, coffee, fruit juices, and sugar-sweetened beverages
HPFS	United States	26	4 670 men	40-75 y	Self-administered SFFQ, every 4 years	Self-report with or without validation with medical records	CVD (1 038)	<1/mo<br>1-<4/mo<br>1-<3/wk<br>3-<5/wk<br>5-<7/wk<br>≥1/d<br>Per 1 egg/d	1.00 (reference)<br>1.06 (0.78, 1.45)<br>1.00 (0.74, 1.36)<br>0.99 (0.71, 1.37)<br>1.05 (0.68, 1.63)<br>1.05 (0.69, 1.61)<br>0.93 (0.75, 1.16)	Age, calendar time, smoking status, BMI, physical activity, family history of myocardial infarction, baseline high blood cholesterol, baseline high blood pressure, alcohol intake, multivitamin use, daily intake of total calories, full-fat milk, bacon, red meat, other processed meats, refined grains, fruits, vegetables, potatoes, coffee, fruit juices, and sugar-sweetened beverages

* Relative risk estimates were obtained from Cox proportional hazards regression models in all studies.

† CHD included coronary artery disease, myocardial infarction, ischemic heart disease; CVD included combined vascular events (CHD and stroke).

‡ The risk estimates from Houston et al. and Trichopoulou et al. among individuals with diabetes were included in the meta-analysis of studies among the general population as no estimate was available for the entire cohort.

BMI: body mass index; CHD: coronary heart disease; CVD: cardiovascular disease; FFQ: food frequency questionnaire; HDL-C: high-density lipoprotein cholesterol; HPFS: Health Professionals' Follow-up Study; LDL-C: low-density lipoprotein cholesterol; NHANES: National Health and Nutrition Examination Survey; NHS: Nurses' Health Study; SD: standard deviation; SFFQ: semiquantitative food frequency questionnaire; TG: triglyceride.

Supplemental Table 11: List of confounders among studies included in the meta-analysis.

	Abdollahi et al. 2019	Diez-Espino et al. 2017*	Djousse et al. 2008	Farvid et al. 2017	Goldberg et al. 2014	Guo et al. 2018	Houston et al. 2011*	Jang et al. 2018*	Key et al. 2019	Larsson et al. 2015*†	Mann et al. 1997	Misirli et al. 2012	Nakamura et al. 2004†	Nakamura et al. 2006	Nakamura et al. 2018	Qin et al. 2018	Qureshi et al. 2007*	Sauvaget et al. 2003	Scrafford et al. 2011†	Trichopoulou et al 2006*	van den Brandt et al. 2019	Virtanen et al. 2016	Wang et al. 2016	Xu et al. 2018	Yaemsiri et al. 2012	Zazpe et al. 2011	Zhong et al. 2019	Drouin-Chartier et al., current*†
**Primary confounders**																												
Age	X	X	X	X	X	X	X	X	X	X	X	X	X	X	X	X	X	X	X	X	X	X	X	X	X	X	X	X
Sex	NA	X	NA	X	X	NA	X	X	X	NA	X	X	NA	X	NA	X	X	X	NA	X	X	NA	X	X	NA	X	X	NA
Body mass index	X	X	X	X	X	X	X	X	X	X		X	X	X	X	X	X	X	X		X	X	X		X	X	X	X
Smoking status	X	X	X	X	X	X	X	X	X	X	X	X	X	X	X	X	X	X		X	X	X	X	X	X	X	X	X
Physical activity/ energy expenditure	X	X	X	X	X	X	X	X	X	X		X				X					X	X		X	X	X	X	X
Alcohol intake	X	X	X	X	X	X	X	X	X	X			X	X	X	X		X	X	X	X	X	X	X	X	X	X	X
Total energy intake	X	X		X	X	X	X	X	X	X		X							X	X	X	X			X	X	X	X
**Secondary confounders**																												
Hypertension and/or blood pressure and/or blood pressure-lowering drugs	X	X	X	X	X		X	X	X	X		X	X	X	X	X	X	X	X	X	X	X		X	X	X	X	X
Dyslipidemia and/or lipid profile and/or lipid-lowering drugs		X	X		X		X	X	X	X			X	X	X		X			X				X	X		X	X
Red and/or processed meat intake						X		X	X	X				X	X					X								X
Mediterranean food pattern (including red and/or processed meat)		X			X																							
Dietary pattern (including red and/or processed meat)																X												
**Other confounders**																												
Intakes of:																												
Refined grains/white bread																												X
Coffee																				X								X

Milk/Dairy products									X											X								X
Poultry									X																			
Fruit juices						X														X								X
Fish									X					X						X								
Vegetables	X		X					X	X	X				X						X	X	X			X			
Fruits	X							X	X	X				X						X	X				X			
Berries	X																					X						
Breakfast cereal			X																									
Potatoes																				X								X
Legumes																				X								
Nuts																				X								
Sugar-sweetened beverages																												X
Sugar and confectioneries						X			X											X								
Non-alcoholic beverages																												
Proteins					X		X																					
Fibers						X	X	X	X						X	X						X			X			
Cereals																				X								
Carbohydrates					X																							
Monounsaturated fatty acids (MUFA)																												
Saturated fatty acids (SFA)					X		X															X						
Polyunsaturated fatty acids																						X						
Sodium															X													
Olive oil																				X								
Dietary supplement use								X													X							
Vitamin/multivitamin use			X				X	X																	X			X
Opium use				X																								
Whether participants intended to avoid cholesterol rich diets														X														
Diabetes/blood glucose/treatment for diabetes		X	X		X	X	NA		X	X		X	X	X	X		X	X	X	X	X	X		X	X	X	X	
Aspirin use							X			X						X									X			
Oral estrogen use							X																		X		X	X
Oral contraceptive use																												X
Postmenopausal HRT																					X							
Medication				X																								
Family history of CVD		X			X	X				X						X						X			X	X		X

Family history of cancer				X																								
Social class						X					X																	
City/center/residency/commune		X		X			X	X	X									X					X					
Education		X		X			X	X	X	X		X				X	X	X	X	X	X	X		X	X		X	
Employment status									X															X				
Marital status				X												X			X									
Race/ethnicity				X	X		X										X		X						X		X	X
Season																							X					
Family income/wealth score				X				X								X								X	X			
Height																					X							
Waist-to-hip																X												
Waist																												
Waist-to-height																				X								
Hip circumference																				X								
Treatment arm			X																									
Atrial fibrillation			X																						X			
Premature myocardial infarction			X																									
Cohort effects														X				X										
Examination year	X																					X						
Radiation dose																		X										
Serum creatinine													X															
MET score																				X								
Self-rated health																								X				

* Study includes risk estimates for individuals with type 2 diabetes.

† Study includes risk estimates for men and women separately.

Supplemental Table 12: Assessment of risk of bias with the Newcastle-Ottawa Scale.*

Author, year (Study ID)	Selection				Comparability		Outcome			Total
	Representativeness of the exposed cohort*	Selection of the non-exposed cohort	Ascertainment of exposure	Outcome of interest not present at start of the study	Control for primary confounders	Control for secondary confounders	Assessment of outcome	Duration of follow-up	Adequacy of follow-up	
Abdollahi et al. 2019	1	1	0	1	1	0	1	1	1	7
Díez-Espino et al. 2017†	0	1	1	1	1	1	1	0	1	7
Djousse et al. 2008	0	1	1	1	0	0	1	1	1	6
Farvid et al. 2017	1	1	0	1	1	0	0	1	1	6
Goldberg et al. 2014	1	1	0	1	1	1	1	1	1	8
Guo et al. 2018	1	1	1	1	1	0	1	1	1	8
Houston et al. 2011†	1	1	0	1	1	0	1	0	1	6
Jang et al. 2018*	1	1	1	1	1	1	1	0	0	7
Key et al. 2019	1	1	0	1	1	1	1	1	1	8
Larsson et al. 2015†‡	1	1	0	1	1	1	1	1	1	8
Mann et al. 1997	0	1	0	1	0	0	1	1	1	5
Misirli et al. 2012	0	1	0	1	0	0	1	1	1	5
Nakamura et al. 2004‡	1	1	0	1	0	0	1	1	1	6
Nakamura et al. 2006	1	1	0	1	0	1	1	1	1	7
Nakamura et al. 2018	1	1	0	1	0	1	1	1	1	7
Qin et al. 2018	1	1	1	1	0	0	1	0	1	6
Qureshi et al. 2007†	1	1	0	1	0	0	1	1	1	6
Sauvaget et al. 2003	1	1	0	1	0	0	1	1	1	6
Scrafford et al. 2011‡	1	1	0	1	0	0	1	0	1	5
Trichopoulou et al 2006†	1	1	0	1	0	1	1	0	1	6
van den Brandt et al. 2019	1	1	0	1	1	0	1	1	1	7
Virtanen et al. 2016	1	1	0	1	1	0	1	1	1	7
Wang et al. 2016	0	1	0	1	0	0	1	1	1	5
Xu et al. 2018	1	1	0	1	0	0	1	0	1	5
Yaemsiri et al. 2012	0	1	1	1	1	0	1	0	1	6
Zazpe et al. 2011	0	1	0	1	1	0	1	0	1	5
Zhong et al. 2019	1	1	0	1	1	0	1	1	?	6
Drouin-Chartier et al. current†‡	0	1	1	1	1	1	1	1	1	8

* Representativeness of the exposed cohort: 1 point awarded if community-based population; Selection of the non-exposed cohort: 1 point awarded if drawn form the same community as the exposed cohort; Ascertainment of exposure: 1 point awarded if diet assessed at baseline and at least one time during follow-up; Outcome of interest not present at start of the study: 1 point awarded if individuals with prevalent CVD at baseline were excluded; Control for primary confounders: 1 point awarded if adjustment for age, sex, BMI, smoking status, alcohol intake, and physical activity; Control for secondary confounders: 1 point awarded if adjustment for hypertension and/or blood pressure and/or blood pressure-lowering drugs, and dyslipidemia and/or lipid profiles and/or lipid-lowering drugs, and total energy intake, and at least one dietary confounders that included red and/or processed meat intake (e.g. adjustment for red and/or processed meat intake or adjustment for dietary pattern that included meat); Assessment of outcome: 1 point awarded if non-fatal cases were confirmed by physician's diagnosis, and fatal cases confirmed by death certificates; Duration of follow-up; 1 point awarded if follow-up ≥ 10 years; Adequacy of follow-up: 1 point awarded if loss to follow-up <20%.

† Study includes risk estimates for individuals with type 2 diabetes; ‡ Study includes risk estimates for men and women separately.

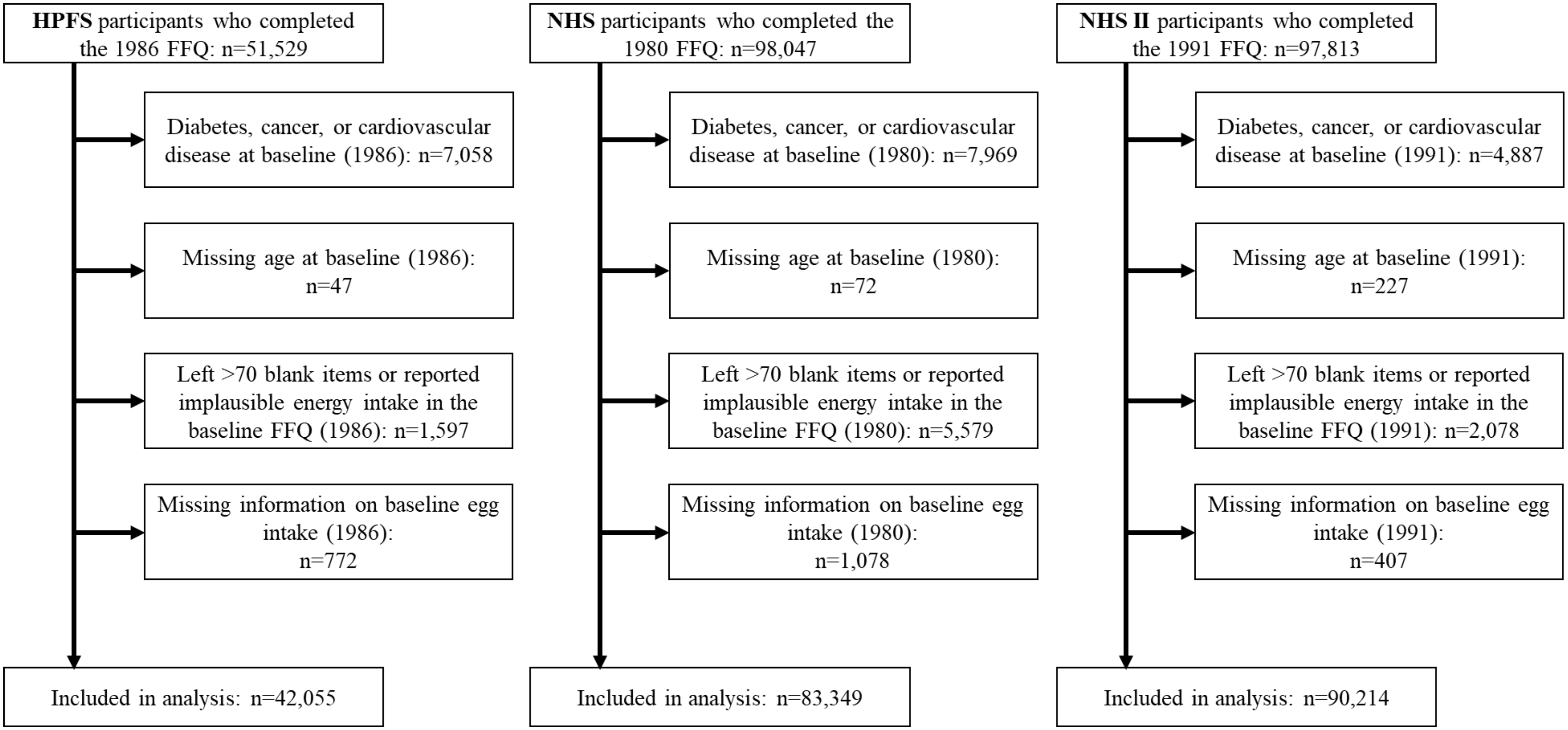

Supplemental Figure 1: Flow chart of participants.

FFQ: Food frequency questionnaire; HPFS: Health Professionals' Follow-up Study; NHS: Nurses' Health Study.

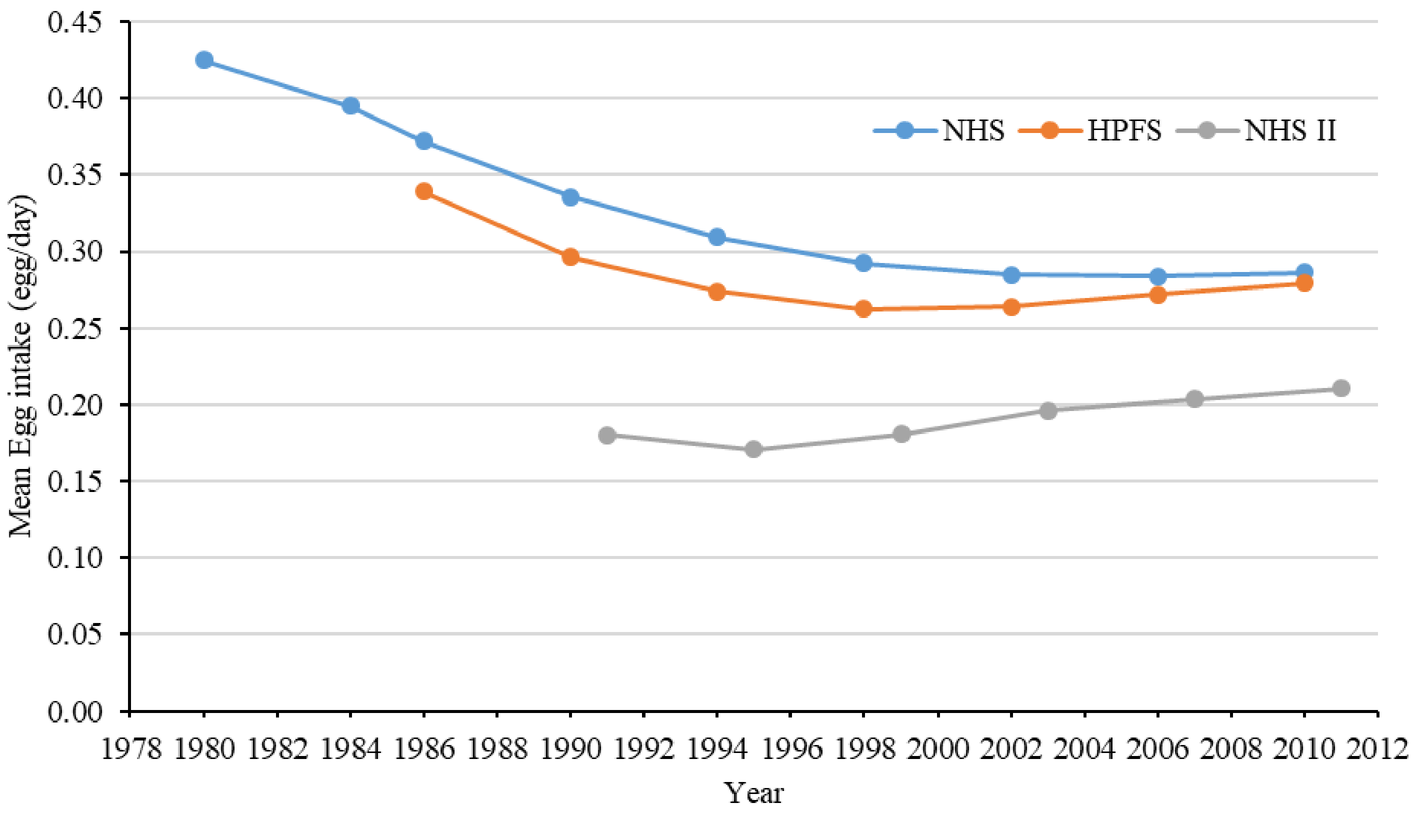

Supplemental Figure 2: Trends in mean egg consumption in the Nurses' Health Study, Nurses' Health Study II, and Health Professionals Follow-Up Study over time.

Mean egg intake calculated for each questionnaire returned.

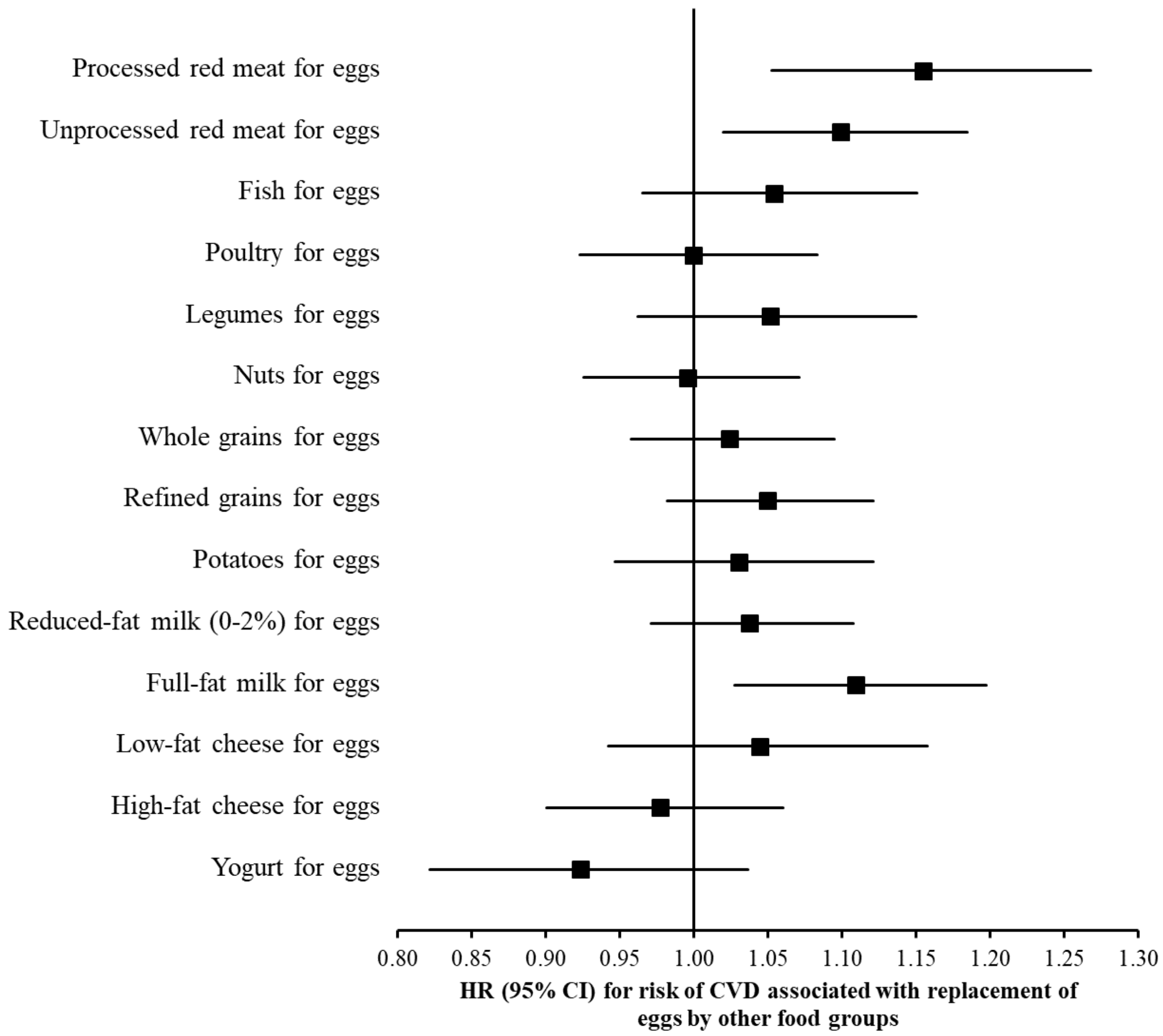

Supplemental Figure 3: Statistical model-based hazard ratios and 95% confidence intervals for incident cardiovascular disease associated with replacement of one egg per day with one serving per day of other foods.

Substitution analyses were stratified by calendar time (in 2-year intervals) and cohort, and adjusted for age (months), race (Caucasian, other), family history of myocardial infarction (yes, no), baseline hypercholesterolemia (yes, no), baseline hypertension (yes, no), smoking status (never, former, current), body mass index (kg/m^2: categorical <21.0, 21.0-22.9, 23.0-24.9, 25.0-26.9, 27.0-29.9, 30.0-34.9, ≥35.0), physical activity (metabolic equivalent of task-hours/week: categorical <3.0, 3.0-8.9, 9.0-17.9, 18.0-26.9, ≥27.0), oral contraceptive use (never, former, current, in NHS II only), postmenopausal hormone use (premenopausal, never, former, current, in NHS and NHS II), alcohol intake (g/d in quintiles), and multivitamin use (yes, no). Model also included updated cumulative average of daily intake of total calories (kcals/d in quintiles), full-fat milk (servings/d in categories), bacon (servings/d in categories), unprocessed red meat (servings/d in categories), other processed meats (servings/d in categories), refined grains (servings/d in categories), fruits (servings/d in categories), vegetables (servings/d in categories), potatoes (servings/d in categories), coffee (servings/d in categories), fruit juices (servings/d in categories), and sugar-sweetened beverages (servings/d in categories). All covariates (except race, family history of myocardial infarction, baseline hypercholesterolemia and hypertension) were updated every 2 years.

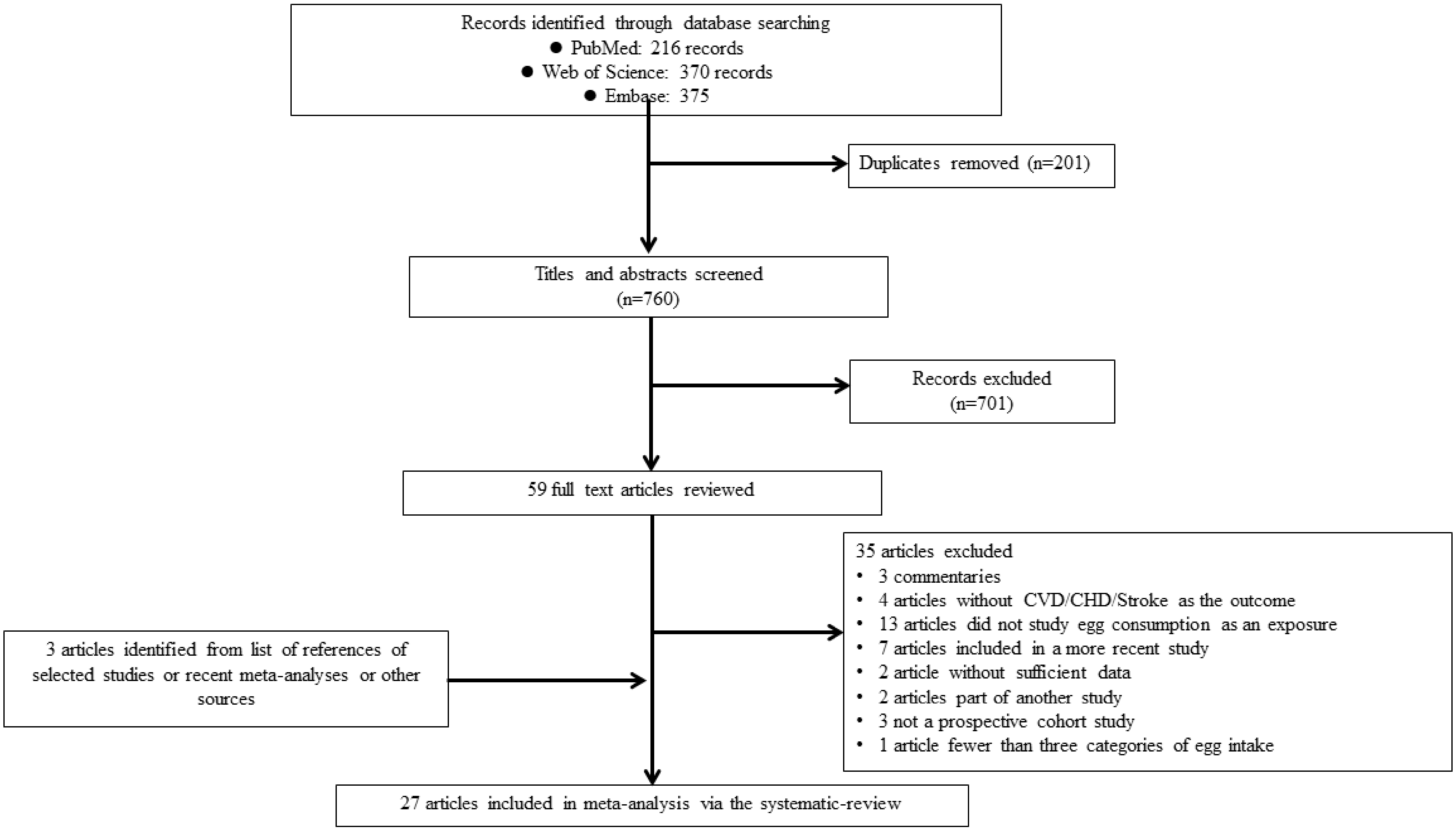

Supplemental Figure 4: Flow chart of study selection.

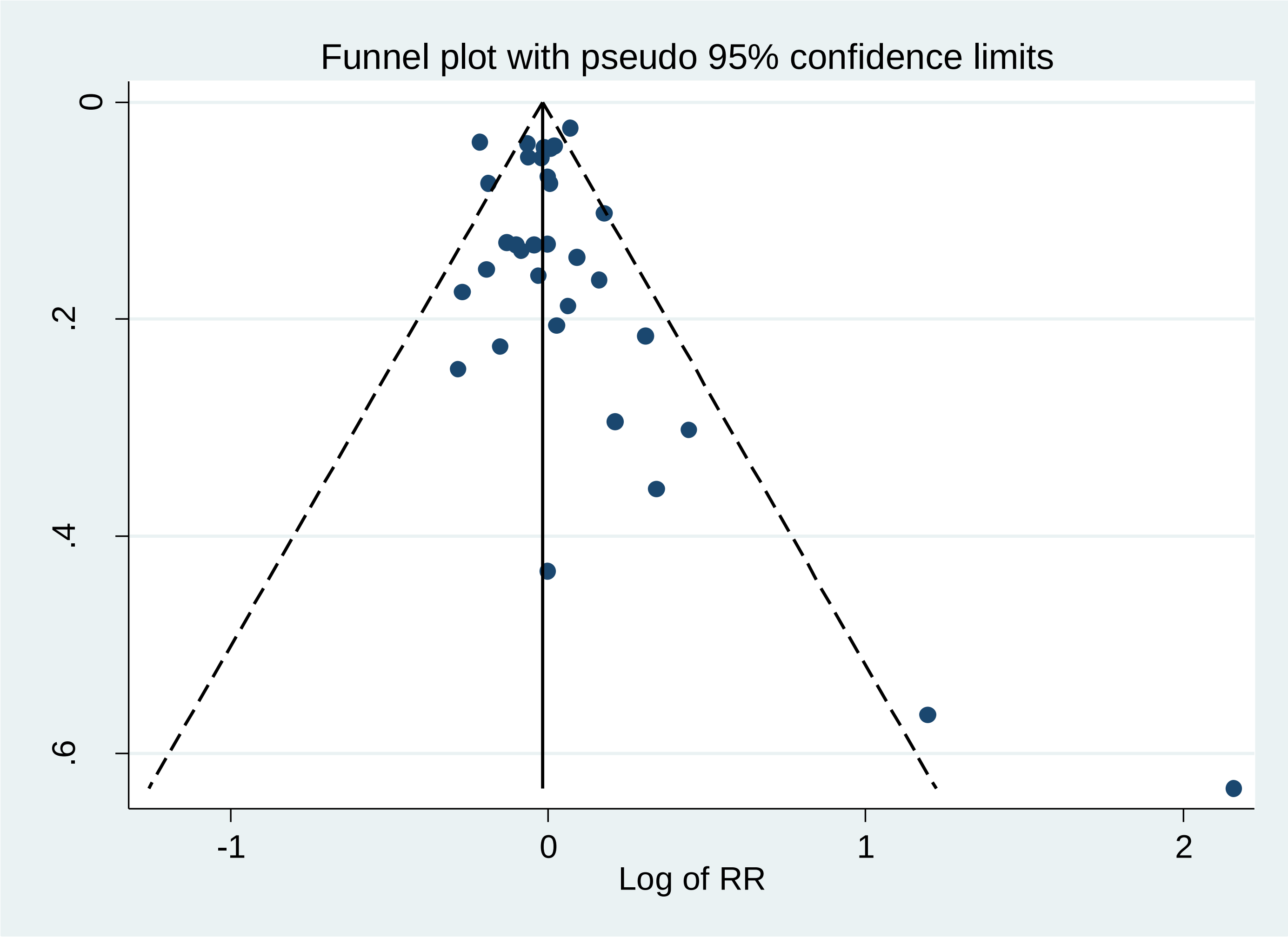

Supplemental Figure 5: Funnel plot for assessment of publication bias for the association between egg consumption and risk of cardiovascular disease.

P value for Egger's test = 0.44; *P* value for Begg's test = 0.08.

CVD: cardiovascular disease; RR: relative risk.

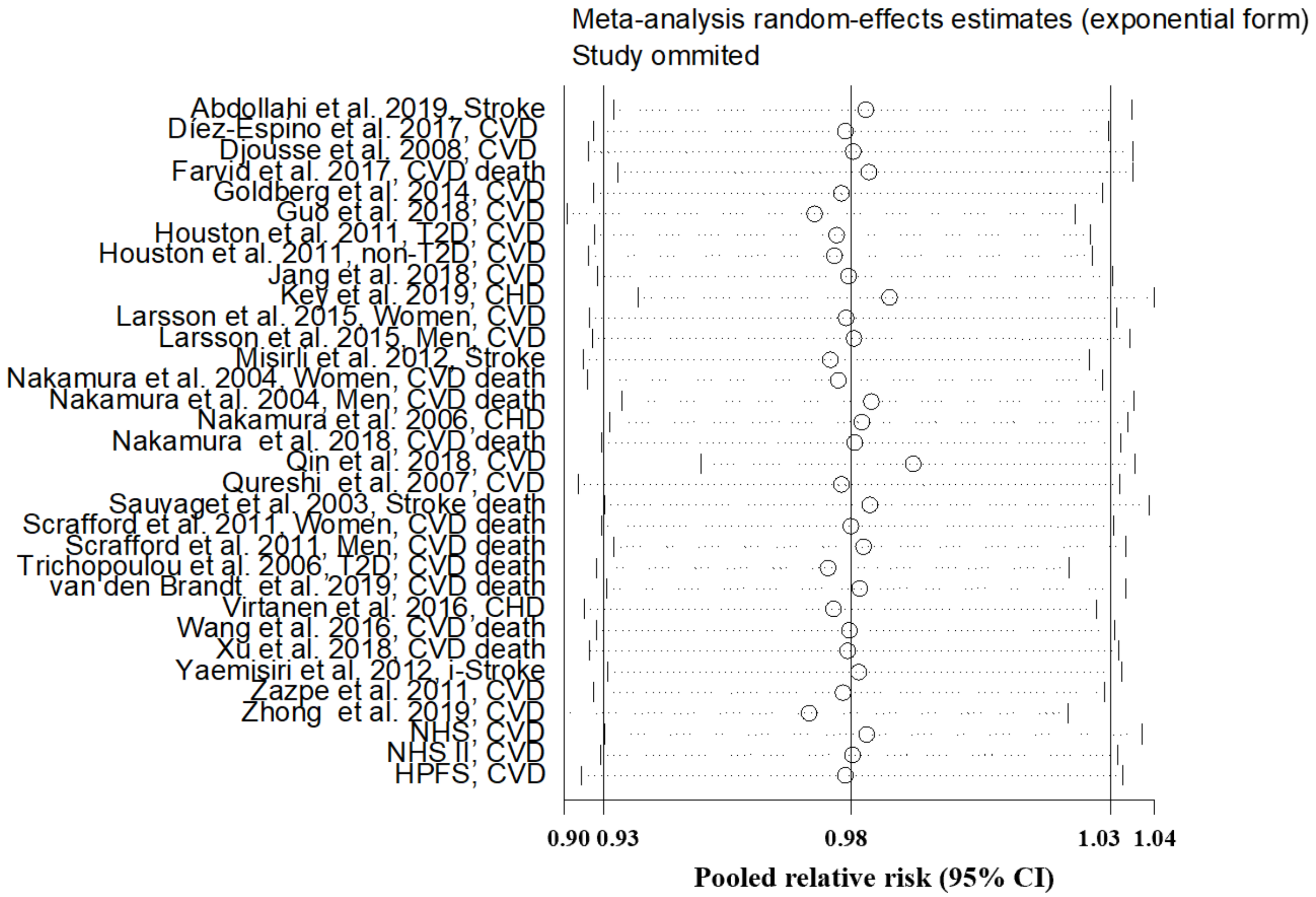

Supplemental Figure 6: Forest plot of influence analysis for the association between egg consumption and risk of cardiovascular disease.

Each dot represents the pooled RR (95% CI) following the exclusion of the study listed on the left using random-effects meta-analysis. CVD: cardiovascular disease; CHD: coronary heart disease; H-stroke: hemorrhagic stroke; I-stroke: ischemic stroke; NHS: Nurses' Health Study; HPFS: Health Professionals Follow-Up Study.
